# A coupled process of same- and opposite-sex mating generates polyploidy and genetic diversity in *Candida tropicalis*

**DOI:** 10.1371/journal.pgen.1007377

**Published:** 2018-05-07

**Authors:** Han Du, Qiushi Zheng, Jian Bing, Richard J. Bennett, Guanghua Huang

**Affiliations:** 1 State Key Laboratory of Genetic Engineering, School of Life Sciences, Fudan University, Shanghai, China; 2 Institutes of Biomedical Sciences, Fudan University, Shanghai, China; 3 State Key Laboratory of Mycology, Institute of Microbiology, Chinese Academy of Sciences, Beijing, China; 4 University of Chinese Academy of Sciences, Beijing, China; 5 Department of Molecular Microbiology and Immunology, Brown University, Providence, Rhode Island, United States of America; Duke University Medical Center, UNITED STATES

## Abstract

Sexual reproduction is a universal mechanism for generating genetic diversity in eukaryotes. Fungi exhibit diverse strategies for sexual reproduction both in nature and in the laboratory. In this study, we report the discovery of same-sex (homothallic) mating in the human fungal pathogen *Candida tropicalis*. We show that same-sex mating occurs between two cells carrying the same mating type (*MTL***a**/**a** or α/α) and requires the presence of pheromone from the opposite mating type as well as the receptor for this pheromone. In ménage à trois mating mixes (i.e., “**a** x **a** + α helper” or “α x α + **a** helper” mixes), pheromone secreted by helper strains promotes diploid *C*. *tropicalis* cells to undergo same-sex mating and form tetraploid products. Surprisingly, however, the tetraploid mating products can then efficiently mate with cells of the opposite mating type to generate hexaploid products. The unstable hexaploid progeny generated from this coupled process of same- and opposite-sex mating undergo rapid chromosome loss and generate extensive genetic variation. Phenotypic analysis demonstrated that the mating progeny-derived strains exhibit diverse morphologies and phenotypes, including differences in secreted aspartic proteinase (Sap) activity and susceptibility to the antifungal drugs. Thus, the coupling of same- and opposite-sex mating represents a novel mode to generate polyploidy and genetic diversity, which may facilitate the evolution of new traits in *C*. *tropicalis* and adaptation to changing environments.

## Author summary

The fungal pathogen *Candida tropicalis* not only lives as a commensal in humans but is also widely distributed in diverse environments. Until recently, *C*. *tropicalis* was thought to be an asexual diploid organism. In this study, we report the discovery of same-sex mating and reveal an unusual process in which same- and opposite-sex mating are coupled in this fungus. The coupling process represents a novel mode of mating which produces unstable polyploid products and results in a high level of genetic and phenotypic diversity. This biological process may benefit the adaptation of *C*. *tropicalis* to a variety of ecological niches and promotes survival under stressful conditions. Our study expands the repertoire of mating strategies in fungi and sheds new lights on the generation of polyploidy and genomic flexibility.

## Introduction

Sexual reproduction drives the evolution of new traits and adaptation to new environments in eukaryotic organisms. Fungi adopt different strategies for sexual reproduction [1, 2]. Many fungal species exhibit opposite- and same-sex mating (or heterothallic and homothallic reproduction, respectively), both in nature and under experimental conditions [3–5]. For example, the human fungal pathogens *Candida albicans* and *Cryptococcus neoformans* undergo both opposite- and same-sex mating in the laboratory [3, 4]. The vast majority of natural isolates of *C*. *neoformans* are of the α mating type and same-sex mating can occur between both isogenic and non-isogenic α strains in a pheromone-dependent manner [3]. In *C*. *neoformans* and *C*. *albicans*, sexual mating and subsequent meiosis or chromosome loss often results in aneuploid forms and the generation of genetic and phenotypic diversity *de novo* [6, 7].

*C*. *albicans* is a diploid organism that undergoes a parasexual reproductive cycle [8]. Over 90% of natural isolates of *C*. *albicans* are heterozygous at the mating type-like (*MTL*) locus and hence carry both *MTL***a** and *MTL*α idiomorphs [9]. To mate, diploid *MTL***a**/α cells must first undergo homozygosis at the *MTL* locus to become an *MTL***a/a** or α/α (**a** or α) strain. In addition, **a** or α cells must switch from the white to the opaque phenotype to become mating-competent [10]. White and opaque cells are two heritable cell types and can maintain their phenotypic states for many generations. White cells are relatively small and round, whereas opaque cells are larger and elongated [11]. The two cell types also differ in global gene expression profiles and mating competency, with only opaque cells being capable of mating efficiently [10, 12, 13]. Opaque **a** cells secrete **a**-pheromone whereas opaque α cells secrete α-pheromone, and these induce the development of mating projections in cells of the opposite mating type [8]. Inactivation of the Bar1 protease, which is required for degradation of α-pheromone in **a** cells of *C*. *albicans*, allows same-sex mating between opaque “**a** x **a**” cells [4]. The presence of white or opaque helper cells with an opposite *MTL* type also promotes same-sex mating between “**a** x **a**” or “α x α” cells of *C*. *albicans* [4, 14]. *C*. *albicans* therefore undergoes same-sex mating when cells are mixed in a “ménage à trois” mode. In *C*. *neoformans*, same-sex mating (or fruiting) between isogenic cells has been proposed to be an important mode of sexual production in nature [3, 15].

*Candida tropicalis* is also an important human fungal pathogen that is a close relative of *C*. *albicans* [16]. While *C*. *albicans* primarily lives as a human commensal, *C*. *tropicalis* is commonly isolated from humans as well as from certain environmental niches such as sea water, soil, and plants, especially in tropical or subtropical areas [16–18]. Although *C*. *tropicalis* is largely susceptible to antifungals such as fluconazole and amphotericin B, development of resistance has been reported, especially in AIDS patients, intensive care units and leukemia patients [19]. Recently, white-opaque switching and a parasexual cycle have been documented in *C*. *tropicalis* [20–22]. Similar to *C*. *albicans*, *C*. *tropicalis* also has a third heritable cell type, namely the “gray” or “hybrid” type, which is distinguishable from the white and opaque cell types. The gray or hybrid cell type exhibits an intermediate-to-high level of mating competency between that of white and opaque cell types [23, 24].

In this study, we report the discovery of same-sex mating and a coupled process of same- and opposite-sex mating in *C*. *tropicalis*. In the presence of pheromone from the opposite mating type, *C*. *tropicalis* cells can undergo same-sex mating and form *MTL* homozygous tetraploid progeny under laboratory culture conditions. To our surprise, the *MTL* homozygous tetraploid products can further mate with diploid cells of the opposite *MTL* type in ménage à trois mating mixes. The latter mating mixes contained two “**a**” strains and one “α” strain (**a** x **a** +α) or two “α” strains and one “**a**” strain (α x α + **a**).This coupling of homothallic and heterothallic mating processes generated high-ploidy (>4N) strains, which exhibited a high degree of genomic instability and subsequently gave rise to numerous genetically and phenotypically diverse offspring. The coupling of same- and opposite-sex mating may represent a novel mode of sexual reproduction in fungi, and could facilitate the rapid evolution of new traits thereby driving adaptation to changing environments.

## Results

### GlcNAc and alkaline pH conditions promote sexual mating in *C*. *tropicalis*

The frequency of white-to-opaque switching is extremely low under regular culture conditions in some natural isolates of *C*. *tropicalis*, and the opaque phenotype is also unstable in some strain backgrounds [20, 21]. We previously demonstrated that both GlcNAc and pH are critical regulators of white-opaque switching and sexual mating in *C*. *albicans* and *C*. *tropicalis* [25, 26]. Here we performed opposite-mating assays between two *C*. *tropicalis* strains (**a** x α) on four different culture media (Lee’s glucose, pH 6.8 and pH 8.5, and Lee’s GlcNAc, pH 6.8 and pH 8.5) at 25°C. As shown in **S1 Fig**, both GlcNAc and alkaline pH conditions increased the mating efficiency of *C*. *tropicalis* cells. Lee’s GlcNAc with pH 8.5 was found to be the optimal culture medium for mating and, unless specified otherwise, this medium was used for most mating experiments in this study.

### Discovery of same-sex mating in *C*. *tropicalis*

**Fig 1:**
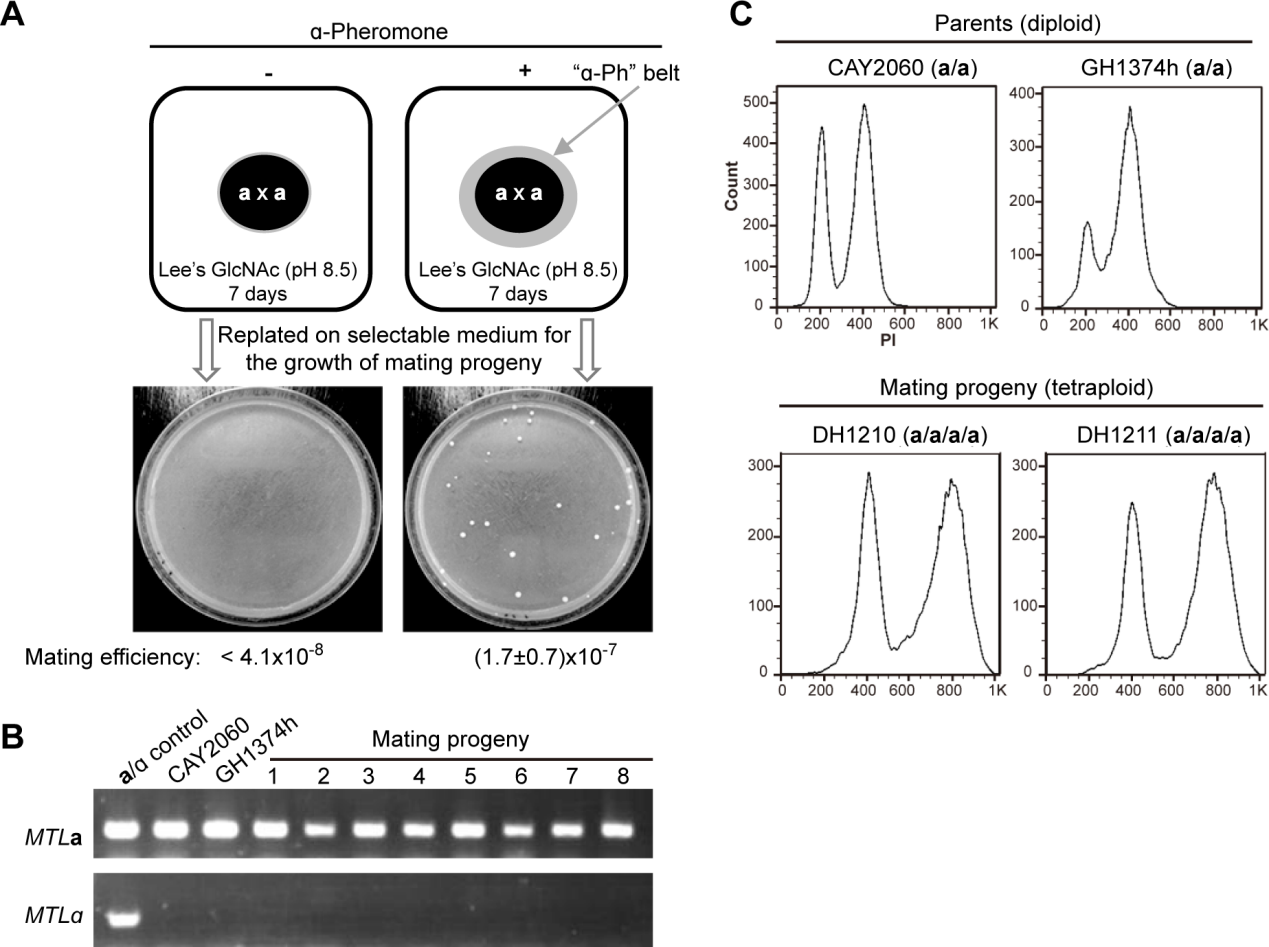
Synthetic α-pheromone induces “a x a” same-sex mating in *C*. *tropicalis*. (A) Cells of two **a** strains (CAY2060, *arg4/arg4*, GH1374h, *his1/his1*) were mixed and spotted on Lee’s GlcNAc medium (pH 8.5) and grown at 25°C for seven days. For the first three days, 40 μL of synthetic α-pheromone (5 mM) was added to the medium surrounding the mating spot every 24 hours. Mock control, no synthetic α-pheromone supplied. After seven days of growth, cells were then replated onto SCD media (-Arg, -His, or -Arg-His) for selection of parental and mating progeny cells. Mating efficiencies were calculated according to the colony numbers obtained from selection media. (B) PCR verification of the *MTL* locus of eight same-sex mating products. An *MTL***a**/α (JX1016) strain and parental (CAY2060, GH1374h) strains served as controls. (C) FACS analysis of two representative mating progeny for genomic DNA content. Parental strains (CAY2060, GH1374h) served as diploid controls.

The presence of pheromone from the opposite mating type can induce the expression of both **a**- and α-pheromone-encoding genes, *MFA1* and *MFα*, respectively, and promote same-sex mating in *C*. *albicans* [4, 14, 27]. Given the genetic and morphological similarities between *C*. *tropicalis* and *C*. *albicans*, we predicted that *C*. *tropicalis* cells would similarly be capable of same-sex mating. We therefore tested this possibility using two “**a**” strains of *C*. *tropicalis*, that are histidine or arginine auxotrophs (*his1/his1* or *arg4/arg4*), respectively. As shown in **Fig 1A**, a mixture of two “**a**” strains was spotted onto Lee’s GlcNAc (pH 8.5) medium and treated with or without synthetic α-pheromone. After seven days of growth at 25°C, cells were replated onto histidine and arginine dropout plates to select for same-sex mating products. Progeny colonies grew on the selective plates when **a** cells had been co-incubated with synthetic α-pheromone, whereas no colonies were observed in the mock-treated control (**Fig 1A**). PCR assays verified that all progeny from “**a** x **a**” mating crosses were *MTL***a** cells like the parental strains (**Fig 1B**). Flow cytometry (FACS) analysis confirmed that mating progeny were tetraploid (**Fig 1C**). These results indicate that synthetic α-pheromone induces same-sex mating between two **a** strains of *C*. *tropicalis*.

**Fig 2:**
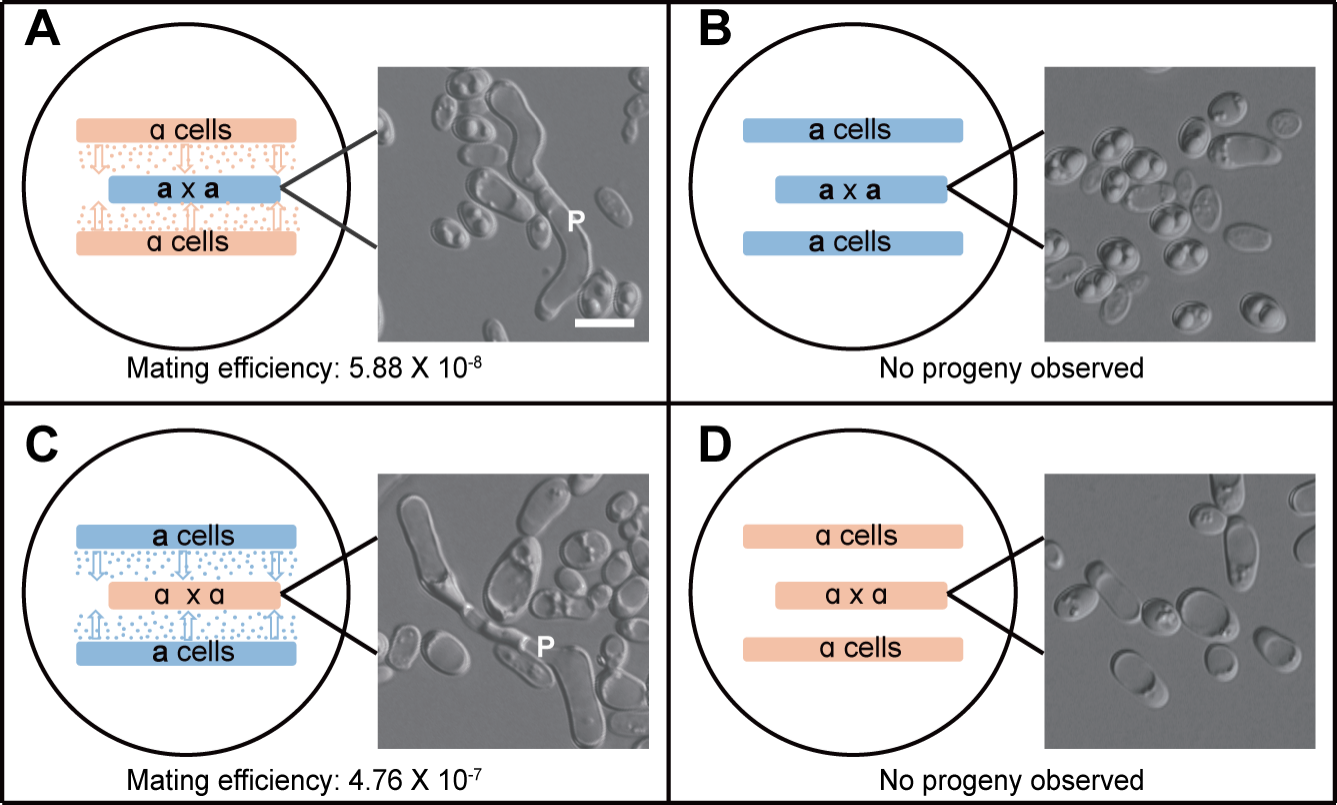
Opposite sex cells induce same-sex mating of *C*. *tropicalis* in a sandwich-culture mode. **a**- or α-cells were patched onto Lee’s GlcNAc medium (pH 8.5) in a sandwich mode as indicated. Cells were cultured at 25°C for seven days. Cells of the “**a** x **a**” or **“**α x α” mating mixture were replated onto selection plates for the growth of parental (SCD-His or -Arg) or mating progeny cells (SCD-His-Arg). Mating efficiencies were calculated and are indicated in the corresponding figures. In the schematics, orange dots represent α-pheromone secreted by helper α cells, and blue dots indicate **a**-pheromone secreted by helper **a** cells. Strains used: “**a** x **a**” mating mixture: CAY2060 x GH1374h; **“**α **x** α” mating mixture: CAY2061 x CAY2063; helper **a** cells: CAY3741; helper α cells: CAY4149. P, mating projection. Scale bar, 10 μM. (A) α cells secrete α-pheromone and induce **“a x a”** same-sex mating. (B) The patches of helper **a** cells serve as the control for (A). (C) **a** cells secrete **a**-pheromone and induce **“**α x α**”** same-sex mating. (D) The patches of helper α cells served as the control for (C). No mating projections were observed in controls (B and D).

A BLAST search revealed that *C*. *tropicalis* has a single ortholog of *C*. *albicans MFα* and has more than 10 copies of the *MFA1* ortholog. We hypothesized that “**a**” cells would secrete **a**-pheromone and thus promote same-sex mating in “α” cells, and *vice versa*. To prove this, we performed “**a** x **a**” or “α x α” same-sex mating assays in a sandwich-culture method (**Fig 2**). Mating progeny were observed arising in the sandwich cultures of both “**a** x **a**” mixtures with α cell side patches and in “α x α” mixtures with “**a**” cell side patches, although mating efficiencies were relatively low (5.9 x 10^−8^ and 4.8 x 10^−7^ for “**a** x **a**” and “α x α” experiments, respectively). However, no progeny were observed in control cultures when using side patches that contained cells of the same *MTL* cell type as in the mating test mixture. These results establish that “**a**” and “α” cells of *C*. *tropicalis* secrete pheromones that promote same-sex mating in cells of the opposite mating type.

**Fig 3:**
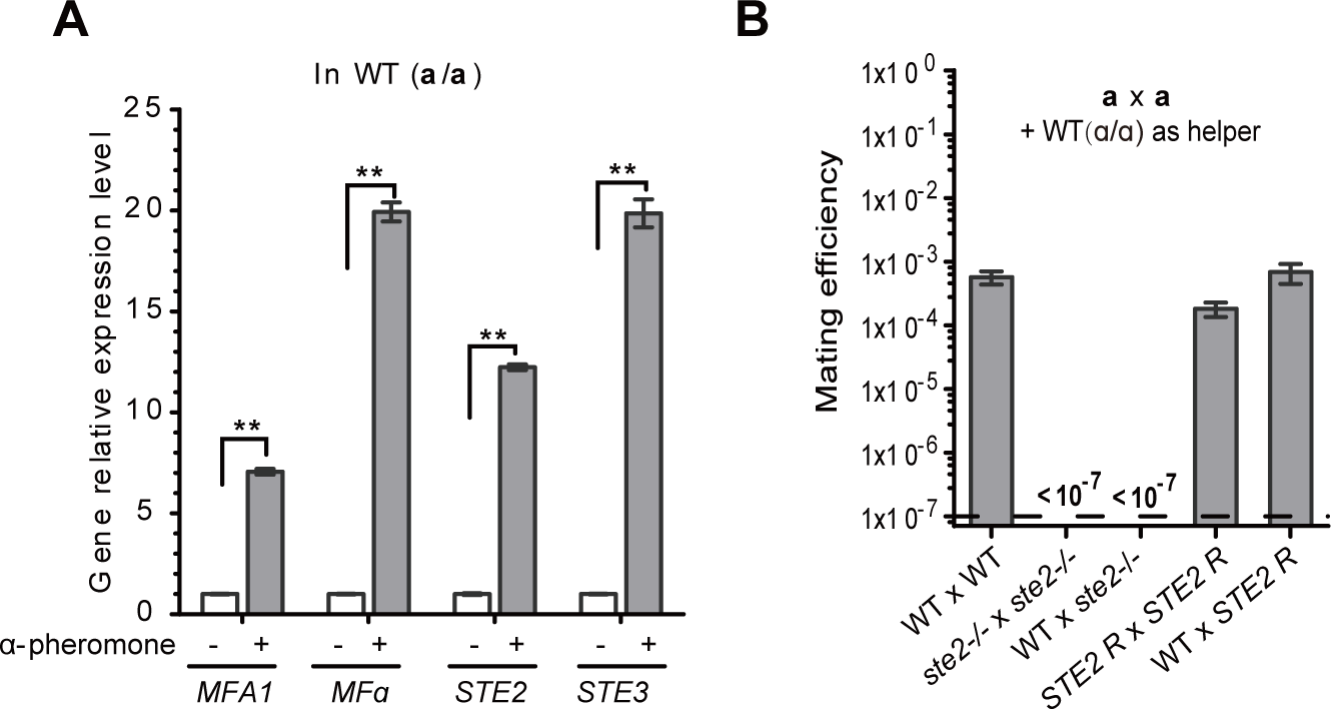
Role of α-pheromone and the Ste2 receptor in same-sex mating in *C*. *tropicalis*. (A) Synthetic α-pheromone induces the expression of *MFA1*, *MFα*, *STE2*, and *STE3* in **a** cells. WT **a** cells were grown on Lee’s GlcNAc medium (pH 8.5) for four days and the resulting opaque cells inoculated into liquid Lee’s GlcNAc medium (pH 8.5) for 48-hour growth at 25°C. Cells were then treated with or without synthetic α-pheromone (100 μΜ) in liquid Lee’s GlcNAc medium (pH 8.5) for six hours. The relative expression levels of *MFA1*, *MFα*, *STE2*, and *STE3* were examined using real-time PCR assays. The expression level of *ACT1* was used for normalization. **, indicates significant difference (P<0.01, two tailed-Student’s *t*-test). Strains used: GH1374h (WT). (B) The α-factor receptor Ste2 is required for “**a** x **a**” same-sex mating when α cells (CAY4149) are present as helper cells. Mating condition: Lee’s GlcNAc medium (pH 8.5) at 25°C for seven days. Strains used: WT x WT: CAY2060 x GH1374h; *ste2/ste2* x *ste2/ste2*: CAY2200 x CAY2202; WT x *ste2/ste2*: CAY2060 x CAY2200; *STE2R* x *STE2R*: CAY2247 x CAY2246; WT x *STE2R*: GH1374h x CAY2246. *STE2R* is a *ste2/ste2* strain in which one copy of *STE2* was been restored.

We next performed quantitative real-time PCR (q-RT-PCR) assays to examine the relative expression levels of *MFA1*, *MFα*, *STE2* (encoding the receptor for α-pheromone), and *STE3* (encoding the receptor for **a**-pheromone) in **a** cells responding to synthetic α-pheromone. As shown in **Fig 3A**, the relative expression levels of the four genes were significantly increased in **a** cells of *C*. *tropicalis* when treated with synthetic α-pheromone. These results indicate that synthetic α-pheromone induces **a** cells to become potential bi-maters that exhibit features of both **a** and α cells in terms of cell identity.

### The α-pheromone receptor Ste2 is required for α cell-induced “a x a” same-sex mating

Ste2 is required for α-pheromone-induced responses in **a** cells and is essential for opposite-sex mating in *C*. *albicans* [28]. To confirm the importance of pheromone signaling, we examined the role of Ste2 in same-sex mating in *C*. *tropicalis* using ménage à trois mating assays (“**a** x **a** + α helper”). As shown in **Fig 3B**, both the WT**a** x *ste2***a** and *ste2***a** x *ste2***a** mating cultures in the presence of WT α cells failed to produce progeny. However, the mating efficiencies of the WT**a** x WT**a** control, WT**a** x STE2R**a**, and STE2R**a** x STE2R**a** were comparable (approximately 1 x 10^−3^; STE2R is a *ste2/ste2* deletion strain in which *STE2* has been reconstituted). These results indicate that the Ste2 receptor is essential for “**a** x **a**” same-sex mating in *C*. *tropicalis*.

### Discovery of coupled same- and opposite-sex mating during ménage à trois cultures

**Fig 4:**
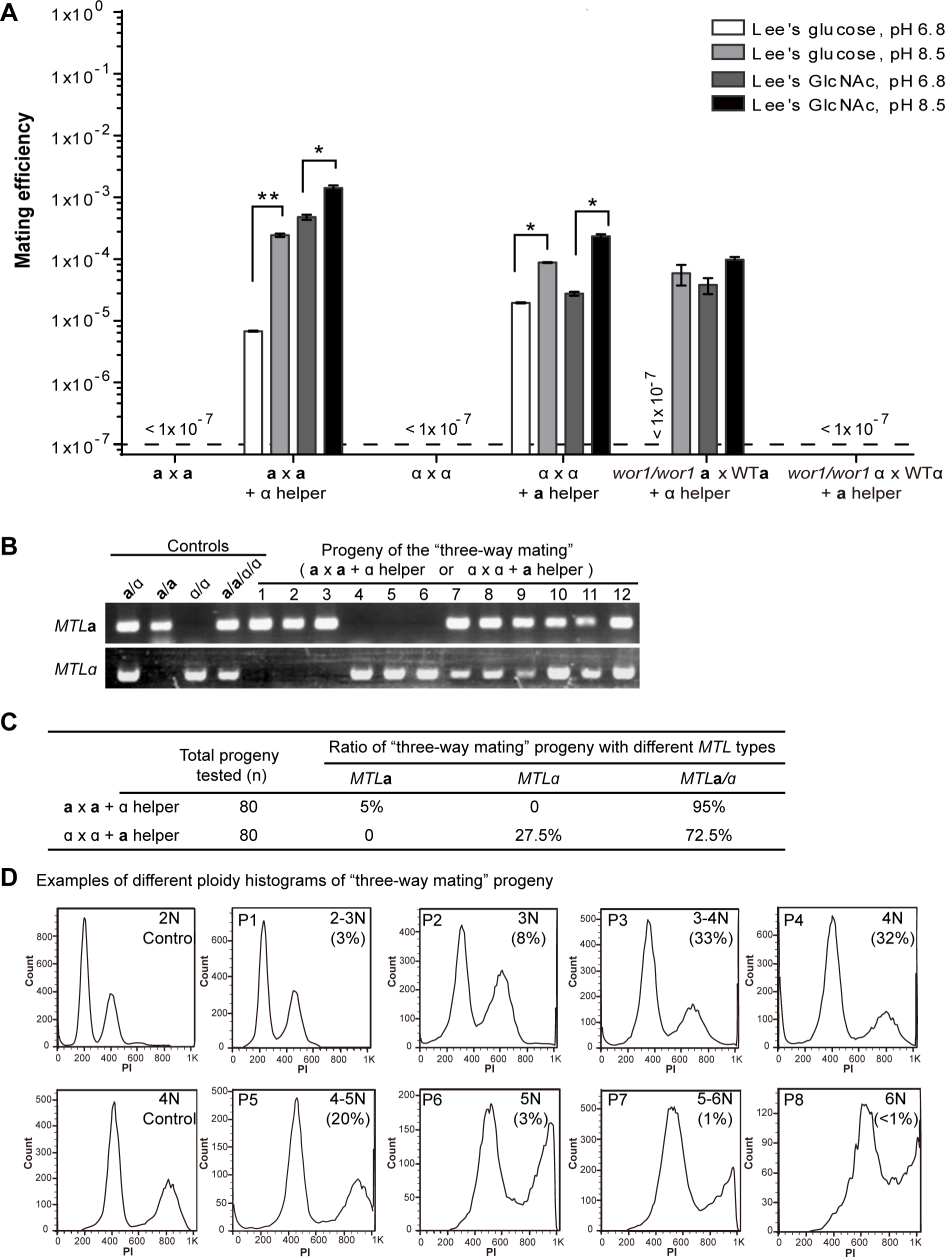
Ménage à trois matings in *C*. *tropicalis*. (A) Efficiency of three-way matings (“**a** x **a** + α helper” or “α x α + **a** helper”). 5 x10^6^ cells of each strain were mixed and cultured on four different media at 25°C for seven days. Cells were then replated onto SCD media (-Arg, -His, or -Arg-His) for selection of parental cells and mating progeny. Mating efficiencies were calculated according to the colony numbers obtained from selection media. “**a** x **a**” or “α x α” mixes without helper cells of the opposite mating type served as controls. Strains used for “**a** x **a** +α helper”: CAY2060 (“**a**”, *arg4/arg4*) or CAY2205 (“**a**”, *arg4/arg4 wor1/wor1*) x GH1374h (“**a**”, *his1/his*) + CAY4149 (“α”, *his1/his1 arg4/arg4*); strains used for “α x α + **a** helper”: CAY2061 (“α”, *his1/his1*) or CAY2342 (“α”, *his1/his1 wor1/wor1*) x CAY2063 (“α”, *arg4/arg4*) + CAY3741 (“**a**”, *his1/his1 arg4/arg4*). <1 x 10^−7^, indicates no mating progeny observed. (B) PCR verification of the *MTL* configurations of mating progeny. Nos. 1, 2, 3, 7, 8, and 9 were progeny from the “**a** x **a** + α helper” mating, whereas Nos. 4, 5, 6, 10, 11, and 12 were progeny from the “α x α + **a** helper” mating. Controls: **a**/α diploid (JX1016), **a**/**a** and α/α (parental strains CAY2060 and CAY2061), and **a**/**a**/α/α tetraploid (DH1175, generated from an **a** x α mating). (C) Ratio of progeny with different *MTL* configurations from the three-way mating assays on Lee’s GlcNAc (pH 8.5). In total, 80 progeny strains were examined for each mating culture. (D) Examples of different ploidy histograms of progeny generated from the “**a** x **a** +α helper” three-way mating. The numbers in brackets indicate the percentages of mating progeny with a certain ploidy or within a certain DNA content range. Totally, 80 progeny strains were examined. Diploid strain (CAY2060) and tetraploid strain from **a** x α mating (DH1175) served as controls.

As demonstrated earlier, the efficiency of same-sex mating induced by synthetic pheromone or opposite mating type cells in a sandwich culture was relatively low (**Figs 1 and 2**). We speculated that the pheromone added to the medium or secreted by opposite mating type cells in the sandwich culture method is rapidly degraded by cells in the mating mixture and not enough to support efficient same-sex mating. We therefore performed traditional ménage à trois mating assays in which cells of different mating types are mixed with one another, as described in **Fig 3B** and previous publications [4, 14]. We grew the three-way mating mixtures (“**a** x **a** + α helper” or “α x α + **a** helper”) on four different media (Lee’s glucose, pH 6.8 and pH 8.5, and Lee’s GlcNAc, pH 6.8 and pH 8.5) at 25°C. The parental mating strains contained a complementary *his1/his1* or *arg4/arg4* nutritional markers, whereas the helper strains were auxotrophic for both histidine and arginine (*his1/his1 arg4/arg4* strains). Consistent with our results of opposite-sex mating, the efficiencies of the three-way matings on Lee’s GlcNAc medium, pH 8.5, were higher than those on the other three media (**Fig 4A**). Furthermore, the efficiencies in the ménage à trois mating assays were much higher than those of “**a** x **a**” and “α x α” same-sex matings induced by synthetic pheromone or by pheromone secreted by neighboring cells in sandwich experiments (**Figs 1** and **2**).

We performed PCR assays to verify the *MTL* types after mating in the ménage à trois cultures (**Fig 4B**). To our surprise, while there were a small number of *MTL***a** or α homozygotes, we identified many mating progeny with a heterozygous *MTL***a**/α cell type. Upon further examination, we found that approximately 5% of mating progeny from the “**a** x **a** + α helper” experiment were *MTL***a** homozygotes, whereas 95% were *MTL***a**/α heterozygotes. Similarly, 27.5% of progeny selected from the “α x α + **a** helper” mating experiment were *MTL*α homozygotes, whereas 72.5% were *MTL***a**/α heterozygotes (**Fig 4C**). We next analyzed the genomic DNA content of mating products using FACS assays. As shown in **Fig 4D**, the genomic DNA of mating progeny varied from 2N to 6N and was enriched in cells that were 3N to 5N. Approximately 40% of the progeny were euploid (3N, 4N, 5N, or 6N) with 60% being aneuploid. The occurrence of high ploidy (>4N) and *MTL***a**/α heterozygotes implies that the *MTL* homozygous products of same-sex mating between “**a** x **a**” or “α x α” cells subsequently underwent an efficient opposite-sex mating with the *MTL* helper strain in the ménage à trois cultures. For example, in the “**a** x **a** + α helper” experiment, the helper strain secreted sufficient pheromone to promote “**a** x **a**” same-sex mating, which generated tetraploid **a/a/a/a** (*HIS*^+^*LEU*^+^) progeny. The tetraploid *MTL***a** cells then mated with α helper cells and generated higher ploidy progeny (6N, **S2A Fig**). An analogous process for “α x α + **a** helper” experiments is assumed to have occurred. Based on the high frequency of isolated progeny with a lower ploidy (<6N), we reasoned that the genome of high-ploidy progeny could be extremely unstable.

To confirm whether tetraploid (**a**/**a**/**a**/**a** or α/α/α/α) strains could mate with diploid cells of the opposite mating type, we performed mating assays on Lee’s GlcNAc medium by crossing a tetraploid strain (either **a**/**a**/**a**/**a** or α/α/α/α) with a diploid α/α or **a**/**a** strain. As demonstrated in **S3 Fig**, tetraploid cells mated efficiently with opposite sex diploid cells and generated high-ploidy **a**/α heterozygous cells.

In the ménage à trois cultures, an alternative coupled opposite-sex/opposite-sex mating process could also generate hexaploid progeny. As shown in **S2B Fig**, diploid “**a**/**a**” and “α/α” cells could first mate and generate tetraploid progeny (**a**/**a**/α/α). Tetraploid “**a**/**a**/α/α” cells could subsequently undergo homozygosis at the *MTL* locus and become “**a**/**a**/**a**/**a**” or “α/α/α/α” cells. The “**a**/**a**/**a**/**a**” or “α/α/α/α” cells could then mate with diploid “α/α” or “**a**/**a**” cells to generate hexaploid progeny. However, the genome of tetraploid progeny was relatively stable under our culture conditions. We estimated that tetraploid cells rarely underwent homozygosis at the *MTL* locus and lost both “**a**” or both “α” chromosomes in our ménage-a-trois cultures. To establish this, we performed quantitative mating assays between a tetraploid “**a**/**a**/α/α” strain (mating products of CAY3741 x CAY2061) and a diploid “**a**/**a (**CAY2060**)**” or “α/α (CAY2063)” strain. We found that the mating efficiencies of both “**a**/**a**/α/α x **a**/**a**” and “**a**/**a**/α/α x α/α” crosses were extremely low (~1 x 10^−6^ to 1 x 10^−5^). Therefore, the occurrence of this alternative mating process was very rare in our mating assays (**S2B Fig**). However, this coupled opposite-sex mating process could occur in nature or under certain specific conditions that affect the stability of the chromosome carrying the *MTL* locus.

### Wor1 is required for “α x α” same-sex mating but not required for “a x a” same-sex mating in the presence of opposite mating type “helpers”

Wor1 is the master regulator of white-opaque switching in *C*. *albicans* and *C*. *tropicalis* [29–31]. Since only opaque cells can mate efficiently in “**a** x α” opposite-mating assays, we next examined whether deletion of *WOR1* affected same-sex mating in *C*. *tropicalis*. As shown in **Fig 4A**, deletion of *WOR1* in α cells blocked same-sex mating between “*wor1/wor1* α x WTα” in the presence of “**a** helper”; however, deletion of *WOR1* in **a** cells did not block same-sex mating between “*wor1/wor1* **a** x WT**a**” in the presence of “α helper”. We do note, however, that the mating efficiencies of the “*wor1/wor1* **a** x WT**a**” cross were only ~10% of those of the “WT**a** x WT**a**” control cross. These results suggest that white-to-opaque switching is essential for “α x α” same-sex mating but not for “**a** x **a**” same-sex mating in *C*. *tropicalis*. The mechanism of phenotypic switching-independent mating needs to be further investigated.

### Morphological diversity of progeny strains generated from coupled same- and opposite-sex mating events

**Fig 5:**
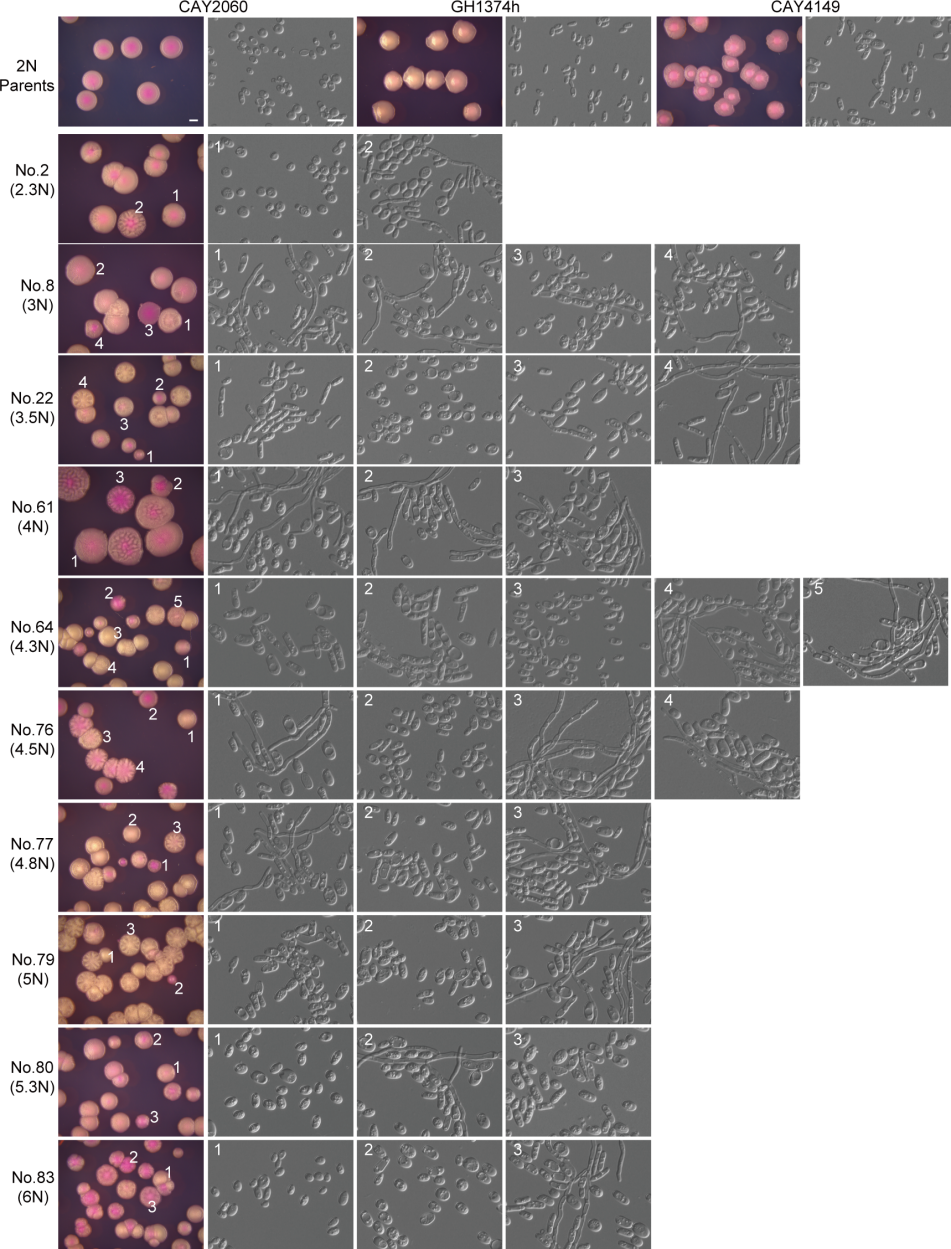
Morphological diversity of progeny generated from the coupled process of same- and opposite-sex mating. Progeny strains with different ploidies from the “**a** x **a** + α helper” experiment were patched onto YPD and incubated at 30°C overnight. Cells were subsequently plated on Lee’s glucose (pH 6.8, with 5 μg/mL of phloxine B) and incubated at 25°C for three days. Colony and cellular images were taken. Colonies with different appearances are numbered and the corresponding cellular morphologies are shown. Diploid parental strains (CAY2060, GH1374h and CAY4149) served as controls. Scale bar for cells, 10 μm; Scale bar for colonies, 1 mm.

The ploidy of progeny from “**a** x **a** + α helper” and “α x α + **a** helper” mating assays varied from 2N to 6N (**Fig 4D**). Genomic changes often lead to phenotypic variation, and morphological change is necessary for virulence in pathogenic *Candida* species [32]. We selected a portion of mating progeny strains with different ploidy levels and performed a morphological analysis on Lee’s glucose medium at 25°C. This culture condition normally does not favor filamentous growth in natural strains of *C*. *tropicalis*. As demonstrated in **Fig 5**, colonies of the three parental strains (CAY2060, GH1374h, and CAY4149) were smooth and contained only yeast cells. However, mating progeny with different genomic DNA content exhibited 2–7 colony types under the same culture condition (**Fig 5**). These colony types included smooth, wrinkled, star-like, and irregular colonies. Some colonies also varied in size, suggesting that growth rates may be different. Microscopy assays demonstrated that different types of colonies contained different cell types including regular yeast-form, opaque and gray-like cells, as well as filamentous cells. For example, progeny No. 22 exhibited at least four types of cellular phenotypes including gray-like (1), white (2), opaque-like (3), and filamentous (4) cells (**Fig 5**). The red dye phloxine B was added to the medium and revealed extensive differences in colony coloration between progeny, which might reflect differences in the structure or integrity of the cell wall (e.g., see #22, 64, and 77).

### Mating progeny-derived strains exhibit distinct antifungal susceptibility

**Fig 6:**
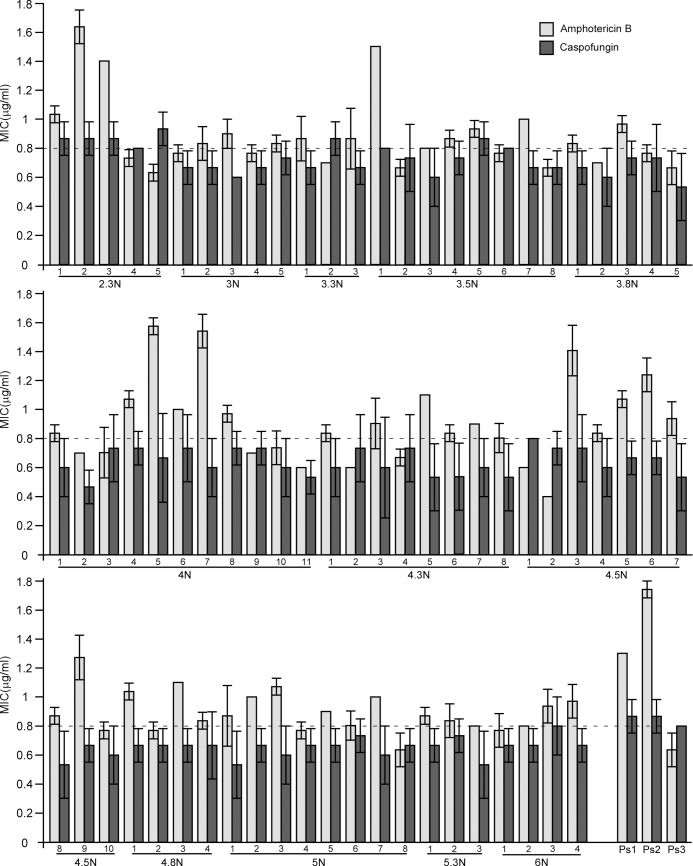
Progeny generated from “a x a +α helper” three-way matings showed diverse responses to amphotericin B and caspofungin. The MIC value was determined in 74 progeny-derived strains with distinct colony appearances from the “**a** x **a** + α helper” mating. Diploid parental strains Ps1, Ps2, and Ps3 (CAY2060, GH1374h and CAY4149) served as controls. MIC assays were performed in 96-well plates. Different wells contained 200 μL of RPMI-1640 medium with amphotericin B or caspofungin in a series of concentrations (from 0.3 to 2.4 μg/mL). Three biological replicates were performed and the average values are presented. Error bars represents corresponding standard deviations.

Genomic variations (especially the occurrence of aneuploidy) often drive the evolution of antifungal resistance in fungi [6, 7, 33]. We next tested the susceptibility to amphotericin B (Amp) and caspofungin (Casp), two potent and widely used antifungal drugs, in 77 representative mating progeny-derived strains selected according to their colony morphologies (as exhibited in **Fig 5**). As shown in **Fig 6**, the minimum inhibitory concentrations (MIC) of Amp for the parental strains (CAY2060, GH1374h, and CAY4149) were about 1.3 μg/mL, 1.8 μg/mL, and 0.7 μg/mL, respectively. Although values varied considerably, all progeny-derived strains tested exhibited a MIC value between 0.4 to 1.8 μg/mL, with no strains showing a MIC value higher than 1.8 μg/mL (that of GH1374h). Similar results were observed in the Casp treatments (**Fig 6**) as progeny-derived strains exhibited a MIC value of Casp between 0.4 to 1.0 μg/mL.

### Mating progeny-derived strains exhibit distinct secreted aspartic proteinase (Sap) activities

**Fig 7:**
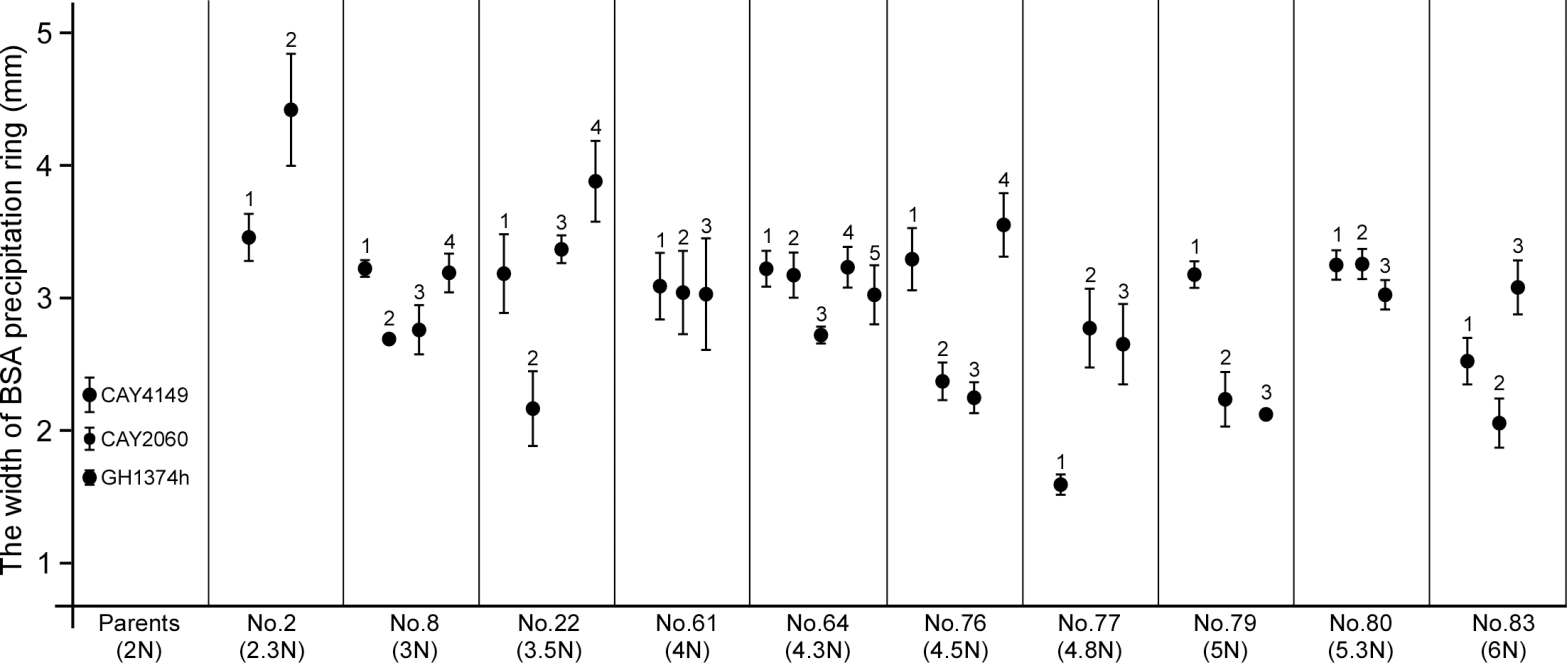
Progeny generated from the coupled process of same- and opposite-sex mating exhibit variable secreted aspartic protease (Sap) activity. The relative Sap activity was quantified by the average value of the width of the BSA precipitation ring (at three representative locations). 37 progeny-derived strains with distinct colony appearances from the “**a** x **a** + α helper” mating were examined. Diploid parental strains (CAY2060, GH1374h and CAY4149) served as controls. Cells were first grown on Lee’s GlcNAc (pH 8.5) at 25°C for seven days. 5×10^6^ cells of each strain in 5 μL ddH_2_O were spotted onto YCB-BSA plates for six days.

Saps represent a major virulence factor of *C*. *tropicalis*, as they are critical for invasive growth in the host and for nutritional acquisition [34]. We examined Sap activity using YCB-BSA halo-ring formation assays [35] in 20 strains derived from progeny of the coupled mating process. As shown in **S4 Fig**, the 20 representative strains exhibited highly variable sizes of the BSA precipitation rings, which reflect the levels of Sap activity, after three days of growth on YCB-BSA medium. To further quantify the Sap activity of the mating progeny, we grew cells for six days and examined the width of BSA precipitation rings. As shown in **Fig 7**, the ring width values of the three parental controls showed limited variation, whereas those of the mating progeny-derived strains varied across a much wider range. Several progeny exhibited a lower level of Sap activity than the parental controls, whereas most showed an increased level of Sap activity compared to controls. This result suggests that the Saps are often expressed at a higher level in the high-ploidy progeny-derived strains than in their diploid parental strains. It is also possible that the selected progeny-derived strains for the Sap activity assays were in a different morphological state to control strains (e.g., wrinkled, filamentous, irregular, or opaque-like) and that these changes caused altered Sap activity.

### High-ploidy mating progeny with distinct morphologies varied in their genomic DNA content

The genome of high-ploidy cells is often unstable in yeasts [36]. The phenotypic diversity of mating progeny-derived strains could therefore be a result of genomic instability associated with their high-ploidy content. We examined the DNA content of several colonies with distinct morphologies (see **Fig 5**) and found that the DNA content of different colonies arising from the same mating product were varied and generally contained less DNA than the mating product (**S5 Fig**). These results indicate that high-ploidy progeny cells often underwent chromosome loss when grown on regular culture medium such as Lee’s glucose medium.

### Mating progeny with higher ploidy exhibit an increased level of genomic instability than lower ploidy strains

**Fig 8:**
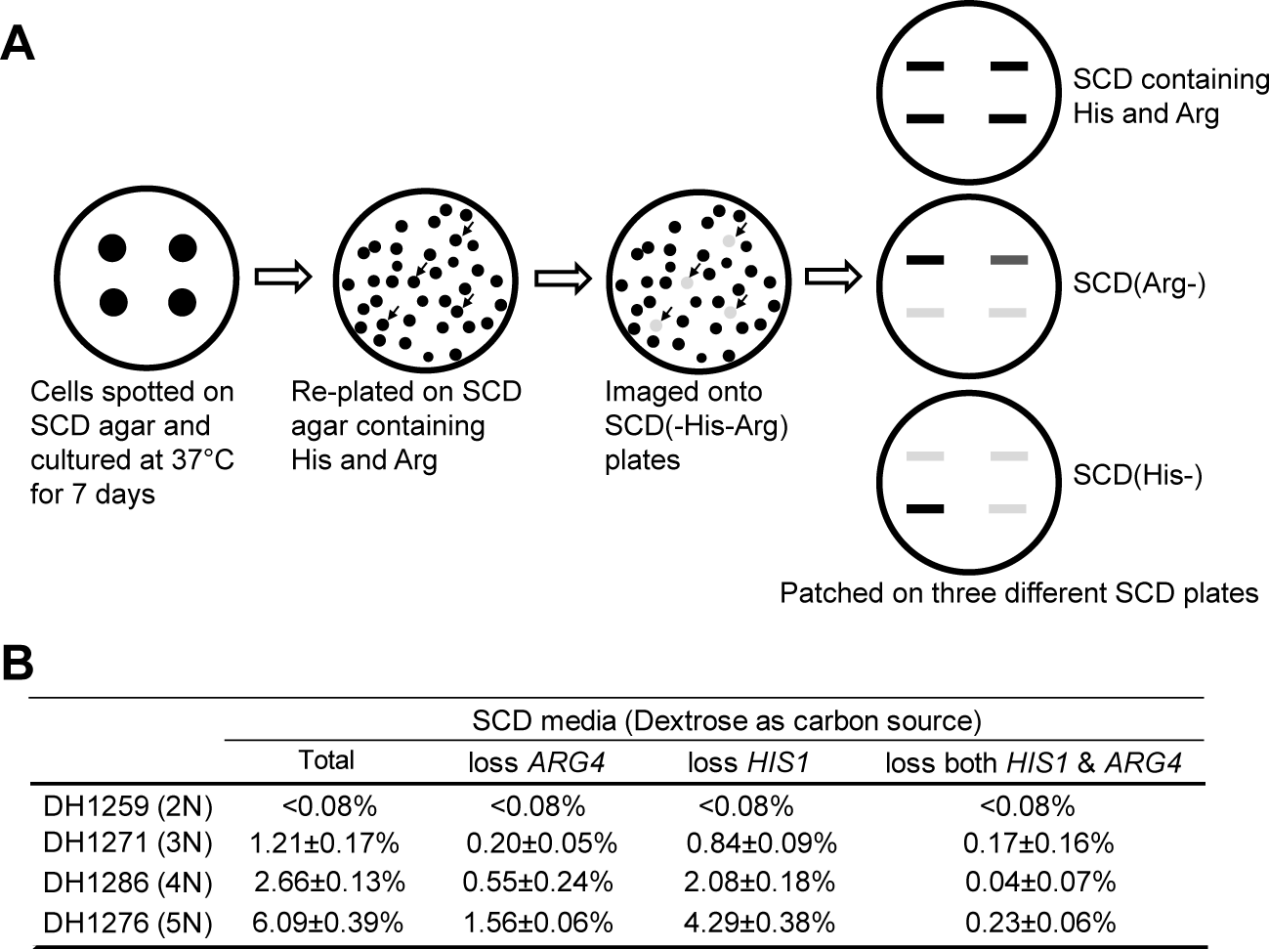
Evaluation of genomic stability using nutritional markers. (A) Schematic of the genome stability assay. Strains with one copy of *HIS1* and one copy of *ARG4* selective markers were generated as described in **Materials and methods**. Replica plating assays were used to determine the presence or absence of *HIS1* and *ARG4*. Colonies that did not grow on the selective plates (-His, -Arg or -both) were patched onto -His or -Arg plates for verification. (B) Frequencies of the loss of *HIS1* and/or *ARG4* nutritional markers in a 2N parental control, and in 3N, 4N, or 5N progeny.

To further elucidate the biological significance of the generation of high-ploidy progeny from coupled same- and opposite-sex mating, we directly tested the genomic stability of mating progeny with 3N, 4N, and 5N genome on SCD medium. These experimental progeny strains were engineered to contain a single copy of *ARG4* and *HIS1* at the endogenous locus. The detailed methods for the generation of these strains are presented in **Materials and methods**. *C*. *tropicalis ARG4* and *HIS1* are on different chromosomes (Butler G., personal communication). Therefore, the frequency of loss of *ARG4* and/or *HIS1* markers could be used as an indicator of genomic instability. As demonstrated in **Fig 8**, the frequencies of marker loss exhibited a gradual increase from 3N progeny to 5N progeny. For example, the frequencies of *ARG4* loss were 0.20±0.05%, 0.55±0.24%, and 1.56±0.06% in the 3N, 4N, and 5N progeny strains, respectively. Considering that, of the 3, 4, or 5 homologous chromosomes in 3N, 4N, and 5N progeny, only one chromosome carried a nutrient marker, the difference between chromosome loss frequencies in cells with different ploidies could be much higher than that presented. These results suggest that genomic instability correlates with increased ploidy in mating progeny.

To further verify whether there would be a tendency to return to lower ploidy levels in high-ploidy progeny, two progeny strains containing 5N or 6N genomic DNA and eight derivatives with a distinct colony morphology were selected for passaging assays. As shown in **S6A Fig**, the ten strains were inoculated into laboratory medium (liquid Lee’s glucose, pH6.8) for 24 hours of growth and then re-inoculated into fresh medium. In total, 20 passages were performed (approximately 130 generations). Original cells and cells of the 5^th^, 15^th^, and 20^th^ passages were subject to genomic DNA analysis. As shown in **S6B** and **S6C Fig**, there was a general tendency for most strains to adopt a lower ploidy. Thus, after 20 passages, most strains stabilized at a lower ploidy state that appeared to be euploid or close to euploid.

## Discussion

In this study, we report the discovery of same-sex mating as well as a coupled process of same- and opposite-sex mating in the fungal pathogen *C*. *tropicalis*. The coupled mating process represents a novel route for the generation of highly polyploid forms of a species. Genetic instability of polyploid cells provides an efficient mechanism for generating genetic and phenotypic diversity, and thereby promotes adaptation to diverse ecological niches and may ensure survival under harsh environments.

Fungi exhibit multiple strategies for sexual reproduction [1]. Similar to its closely related species *C*. *albicans*, *C*. *tropicalis* must first undergo a switch from the default white state to the mating-competent opaque state to mate efficiently [20, 21]. Opposite-sex mating involves two cell types with different *MTL* configurations (namely **a** and α). Opaque **a** cells secrete **a**-pheromone to induce the formation of mating projections in opaque α cells, and vice versa. Two diploid cells of opposite sexes then undergo cell fusion and generate tetraploid cells [8]. In the current study, we observed that the presence of cells with an opposite *MTL* type (or synthetic pheromone) can induce same-sex mating between “**a** x **a**” or “α x α” cells in *C*. *tropicalis* (**Figs 1 and 2**). This is similar to what has been reported for same-sex mating of *C*. *albicans* cells [4, 14]. Interestingly, although white cells of *C*. *albicans* are mating-incompetent, they can be induced to secrete pheromone and thereby help both same- and opposite-sex mating in opaque cells in a ménage à trois mating mixes [14]. In *C*. *tropicalis*, the tetraploid products of same-sex mating retain their opaque cell identity after mating [37] and these progeny (**a**/**a**/**a**/**a** or α/α/α/α) can therefore potentially mate with an opposite-sex strain. We now show that mating of a homozygous 4N cell and an opposite-sex 2N cell occurs very efficiently under ménage à trois mating conditions (95% efficiency for “**a** x **a** + α helper” mixes and 72.5% efficiency for “α x α + **a** helper” mixes, **Fig 4C**). This is because the efficiency of opposite-sex mating is much higher than that of same-sex mating, as described for another fungal pathogen, *C*. *neoformans* [3]. In this coupled two-step mating system, the initial same-sex mating generates tetraploid progeny of *C*. *tropicalis*, which then efficiently mate with the ‘helper’ strain that has a complementary *MTL* type to produce hexaploid progeny. In agreement with this, we observed efficient mating in 4N x 2N opposite-sex mating assays (**S3 Fig**).

FACS analysis revealed that hexaploid progeny were rare in the combined same- and opposite-sex mating experiments in *C*. *tropicalis* (**Fig 4D**), implying that hexaploid progeny were unstable. Even under regular laboratory culture conditions (in Lee’s glucose or SCD medium), polyploid cells underwent rapid chromosome loss and produced extensive genetic variation (**Fig 8B** and **S6 Fig**). Although there was a general tendency towards cells adopting lower ploidy states, some cells with a high genomic content were relatively stable (**Fig 4D and S6 Fig**). Overall, the loss of chromosomes resulted in a number of aneuploid cell states, although a relatively large portion (~40%) of mating progeny were 3N to 5N cells with euploid, or close to euploid, genomes (**Fig 4D**). These results reveal that polyploid cells often undergo concerted chromosome loss to stable cell states with a balanced complement of chromosomes.

It has been demonstrated that specific aneuploid forms of *C*. *albicans* can provide a selective advantage under antifungal stresses [33]. (Para)sex generates high ploidy and promotes genetic diversity and even *de novo* genomic changes in both *C*. *albicans* and *C*. *neoformans* populations [6, 7]. It has been proposed that same-sex mating contributed to the hypervirulent isolates of *Cryptococcus gattii* responsible for the Vancouver Island outbreak that started in 1999 [38]. These studies suggest that sexual reproduction confers novel traits to pathogenic species by producing recombinant progeny that include important genomic changes. These changes can enable fungi to better adapt to new ecological niches. In *C*. *tropicalis*, a higher ploidy (>4N) state generated in the coupled process of same- and opposite-sex mating might have an even more profound impact on generating genetic and phenotypic diversity. To evaluate phenotypic properties, we examined a number of diverse mating progeny for drug resistance, cell morphologies and Sap activity. As shown in **Fig 6**, the MIC values of the antifungal amphotericin B varied from 0.4 to 1.8 μg/mL in different progeny-derived strains. However, none of these strains exhibited a higher resistance to amphotericin B than the most resistant parental strain (GH1374h). Polyploid progeny produced multiple morphological types including opaque-like, gray-like, and filamentous phenotypes even under regular culture conditions (Lee’s glucose medium at 25°C, **Fig 5**). We also examined Sap activity given that this is a major virulence factor in pathogenic *Candida* species [34]. YCB-BSA assays demonstrated that Sap activity varied dramatically among different mating progeny (**Fig 7 and S4 Fig**), and a large portion of strains exhibited higher Sap activity than the parental strains. This could be linked to the morphologies of these strains, as morphological switching and Sap activity are tightly linked with pathogenesis in both *C*. *albicans* and *C*. *tropicalis* [32, 34]. These diversified properties may contribute to the evolution of virulence factors in *C*. *tropicalis* and rapid adaptation to changing environments.

Polyploidy is prevalent in across the tree of life and has been well investigated in model organisms such as the yeast *Saccharomyces cerevisiae* and the plant *Arabidopsis thaliana* [39–41]. The genome of polyploids is also generally unstable in these species. It has been reported that polyploidy can drive rapid adaptation to stressful environments in *S*. *cerevisiae* [41]. In addition, clones evolved from polyploidy exhibited a high frequency of *de novo* mutations [40], which may provide additional selective advantages. However, there could also be some disadvantages to being polyploid. For example, polyploid cells have an increased cell size, changed cellular architecture, and increased inaccuracy of chromosome segregation [39]. In *C*. *albicans*, tetraploids are less virulent and exhibit decreased fitness in the mammalian host [42]. Overall, there is a tendency to return to lower ploidy in each of these species, and a similar phenomenon is observed in *C*. *tropicalis* in our study (**S6 Fig**). Of note, some aneuploid states were relatively stable perhaps due to their increased ability to adapt to certain growth conditions. Moreover, it has been shown that some aneuploid strains were drug resistant and when passaged they lost resistance but only lost some of the aneuploid chromosomes [7]. Chr5 was often stable for example. So, there is a bias for certain chromosomes being more stable.

In summary, together with the previously reported opposite-sex mating, the discovery of same-sex mating in *C*. *tropicalis* indicates that sexual reproduction in pathogenic *Candida* species is conserved in terms of regulatory mechanisms and condition requirements. The coupled process of same- and opposite-sex mating in *C*. *tropicalis* represents a novel route of generating polyploidy, which may accelerate evolution and promote rapid adaptation to changing environments. Here, we therefore provide a new and valuable model with which to investigate the effect of polyploidy and aneuploidy. Our study also sheds new light on the diversified mating modes in fungi and adaptive mechanisms of pathogenic *Candida* species to environmental stresses.

## Materials and methods

### Strains and growth conditions

*C*. *tropicalis* strains used in this study are listed in the supplementary **S1 Table**. Cells were routinely grown in YPD (20 g/L glucose, 20 g/L peptone, 10 g/L yeast extract; 20 g/L agar added for solid medium) or synthetic defined (SD) medium at 30°C. Lee’s glucose (pH 6.8 and pH 8.5) and Lee’s GlcNAc (pH 6.8 and pH 8.5) were used for mating assays. Lee’s glucose (pH 6.8) with the red dye phloxine B (5 μg/mL) was used for morphological assays. For morphological analysis assays, cells were grown on Lee’s glucose (pH 6.8) at 25°C. Colonies and cells were imaged after three days of growth. SD with dextrose as carbon source (SCD) was used for the evaluation of genomic stability. *HIS1* and *ARG4* nutritional markers were used to evaluate the genomic stability of mating progeny. Strains for genomic stability assays were generated as described below. The genome content of all strains used in genomic stability assays was examined by FACS analysis.

#### Parental *MTL*a/a strain (DH1246, *HIS1/his1 arg4/arg4*)

A copy of *HIS1* was re-introduced into the original locus by transforming strain CAY3741 (*MTL***a/a**, *his1/his1 arg4/arg4*) with the *HIS1* gene PCR amplified from *C*. *tropicalis* genomic DNA. All primers used for PCR are listed in **S2 Table**.

#### Parental *MTL*a/a strain (DH1249, *his1/his1 ARG4/arg4*)

A copy of *ARG4* was re-introduced into the original locus by transforming strain CAY3741 (*MTL***a/a**, *his1/his1 arg4/arg4*) with *ARG4* PCR amplified from *C*. *tropicalis* genomic DNA.

#### Parental *MTLɑ*/*ɑ* strain (DH1251, *HIS1/his1 arg4/arg4*)

A copy of *HIS1* was re-introduced into the original locus by transforming strain CAY4149 (*MTLɑ*/*ɑ*, *his1/his1 arg4/arg4*) with the *C*. *tropicalis HIS1* gene.

#### Parental *MTLɑ*/*ɑ* strain (DH1254, *his1/his1 ARG4/arg4*)

A copy of *ARG4* was re-introduced into the original locus by transforming strain CAY4149 (*MTLɑ*/*ɑ*, *his1/his1 arg4/arg4*) with *HIS1* PCR amplified from *C*. *tropicalis* genomic DNA.

#### Diploid control (DH1259, *MTLa*/*a HIS1/his1 ARG4/arg4*)

A copy of *HIS1* was re-introduced into the original locus by transforming strain DH1249 (*MTL***a**/**a**, *his1/his1 ARG4/arg4*) with *ARG4* PCR amplified from *C*. *tropicalis* genomic DNA.

#### Progeny strains of ménage à trios matings (DH1271, DH1286, and DH1276)

Generated from the coupled process of same- and opposite-sex mating of “DH1251 x DH1254 + CAY3741”.

### Mating assay

Opaque cells were used for mating assays. For some strains, if the opaque phenotype was not stable, cells were initially grown on Lee’s GlcNAc (pH 8.5) medium at 25°C for four days. This culture condition is conducive for formation of the opaque phenotype.

Synthetic α-pheromone (KFKFRLTRYGWFSPN) used for induction of same-sex mating in **a** cells of *C*. *tropicalis* was synthesized by the company Scilight-peptide Inc. (Beijing, China). To test α-pheromone-induced same-sex mating in *C*. *tropicalis*, 5 x 10^6^ cells of CAY2060 (*MTL****a***/***a*** *arg4/arg4*) and 5 x 10^6^ cells of GH1374h (*MTL****a***/***a*** *his1/his1*) were mixed, spotted on Lee’s GlcNAc (pH 8.5) plates, and grown at 25°C for seven days. In the first three days, 40 μL synthetic α-factor (5mM) was added to the medium surrounding the mating spots every 24 hours. The mating mixture with no pheromone treatment served as the mock control. After seven days of growth, cells were then replated onto synthetic media (-Arg, -His, or lacking both) for selectable growth of parental and mating progeny cells. Mating efficiencies were calculated according to the colony numbers obtained from SCD media.

To perform the sandwich mating assays, **a**-strains (CAY2060 and GH1374h) or α strains (CAY2061 and CAY2063) were initially grown on Lee’s GlcNAc medium (pH 8.5) for four days at 25°C. Cells from the two **a**-strains (or α-strains) were taken from single colonies, mixed together and patched onto the same medium. Cells with an opposite *MTL* type were then patched close to each side of the same-sex mating mixture in a sandwich mode (as shown in **Fig 2**). The plates were cultured at 25°C for seven days. Cells of the “**a** x **a**” or **“**α **x** α” mating mixture were then replated onto selectable plates for the growth of parental (-His or -Arg) or mating progeny cells (-His -Arg).

To perform ménage à trois mating assays, cells of the parental strains were initially cultured on Lee’s GlcNAc plates (pH 8.5) for four days at 25°C. 5 x 10^6^ of cells of each mating partner and the helper strain (“**a** x **a** + α helper” or “α x α + **a** helper”) were mixed and spotted on Lee’s glucose or Lee’s GlcNAc for seven days of growth at 25°C. The mating mixture was then replated on SCD media (-Arg, -His, or -Arg-His) for selectable growth of parental and mating progeny cells. Mating efficiencies = the greater of (number of progeny/number parent–progeny).

### Quantitative flow cytometry (FACS) analysis

Cells were incubated with shaking in liquid SCD medium. Cultures were harvested, washed, and resuspended in 1 x TE buffer (10 mM Tris, 1 mM EDTA, pH 8.0) and then fixed with 70% ethanol for two hours at room temperature. Cells were then washed with 1 x TE buffer and treated with RNase A (1 mg/mL) for 24 hours and subsequently with proteinase K (5 mg/mL) for two hours at room temperature. Cells were collected, washed with TE buffer, and stained with propidium iodide (PI, 25 μg/mL). Stained cells were washed and resuspended in 1 x TE buffer for DNA content analysis. A total of ~10,000 cells of each strain were run on a FACS Caliber (a multi-Gaussian cell cycle model, BD) and the data was analyzed using the software FlowJo 7.6.1.

### Quantitative real-time PCR assay (RT-qPCR)

RT-qPCR assays were performed as described in our previous publication [26]. Briefly, after treatment with ɑ-pheromone (final concentration 100 μM) in liquid Lee’s GlcNAc (pH 8.5) at 25°C for 6 hours, cells were harvested and washed with ice-cold ddH_2_O. Cells pellet was then subjected to total RNA extraction by GeneJET RNA Purification Kit (Thermo Fisher) following the instruction of manufacture. Total RNA (0.8 μg) was used to synthesize cDNA with RevertAid H Minus Reverse Transcriptase (Thermo Scientific, Inc.) and subject to qPCR assays using SYBR green Mix (TOYOBO, Inc.). The relative expression levels were determined using a Bio-Rad CFX96 real-time PCR detection system and normalized to that of *C*. *tropicalis ACT1*.

### Genomic instability assay

Cells of the control and mating progeny with different ploidy (2N to 5N) were taken from a single colony initially patched on Arg/His dropout plates (SCD-His-Arg) at 25°C for growth overnight. Cells were then collected and washed with ddH_2_O. Approximately 200 cells were spotted on SCD medium containing His and Arg for seven days of growth at 37°C. To examine the loss of *HIS1* or *ARG4*, as an indicator of genomic instability, we tested the frequency of formation of auxotrophic cells (His*-*, Arg*-*, or His*-*Arg-). Briefly, cells from the spot cultures were replated onto SCD medium containing Arg and His and grown at 30°C for 36 hours. The plates were then cultured on Arg-His- dropout SCD plates. The auxotrophic type was verified by patching assays using dropout SCD plates (His*-*, Arg*-*, or His*-*Arg-). Percentage of *his1-* or *arg4-* cells = (number of *his1-* or *arg4-* colonies/total number of colonies) x 100%.

### Secreted aspartyl proteinase (Sap) activity assay

Sap activity was tested using the YCB-BSA method as described previously [35, 43]. Cells of *C*. *tropicalis* were initially grown on Lee’s GlcNAc (pH 8.5) at 25°C for seven days. 5×10^6^ cells of each strain in 5μL ddH_2_O were spotted onto YCB-BSA plates and cultured at 25°C for six days. The width of BSA precipitation rings (halos), which reflect the activity of Saps, was examined at the third and sixth day.

### Minimum inhibitory concentration (MIC) assay

MIC assays were performed according to the NCCLS document M27-A2 and previous publications [7, 44]. Three biological replicates were performed. *C*. *tropicalis* cells of each strain were initially patched on SCD solid medium for 24 hours at 25°C. Approximately, 500 cells were then inoculated into 200 μL RPMI-1640 medium (w/v, 1.04% RPMI-1640, 3.45% MOPs, NaOH used for pH adjustment to 7.0) in a 96-well plate for MIC testing. A series of amphotericin B or caspofungin concentrations (from 0.3 to 2.4 μg/mL) were used. Cells were incubated at 37°C in air for four days. The growth states of cells at different amphotericin B and caspofungin concentrations were recorded.

## Supporting information

S1 Fig Mating efficiency of “a x α” opposite-sex mating on different culture media in *C*. *tropicalis*. Lee’s glucose (pH 6.8 and pH 8.5) and Lee’s GlcNAc (pH 6.8 and pH 8.5) media were used. Strains used: CAY2060 (*MTL***a**) and CAY2061 (*MTL*α). “**a**” cells (5 x10^6^) and “α” cells (5 x10^6^) were mixed and grown on different media at 25°C for seven days. Cells were then replated onto SCD media (-Arg, -His, or -both) for selectable growth of parental and mating progeny cells. Mating efficiencies were calculated according to the colony numbers obtained from SCD media.
(TIF)

S2 Fig Two models for the generation of hexaploidy in ménage à trois cultures of *C*. *tropicalis*. (A) Model for the same- and opposite-sex mating coupled process. Diploid “α/α” cells secrete α-pheromone and promote “**a/a** x **a/a**” same-sex mating. The tetraploid “**a/a/a/a**” progeny cells then mate with diploid “α/α” cells and generate hexaploid progeny. (B) Model for the two consecutive opposite-sex matings. Diploid “**a/a**” cells first mate with diploid “α/α” cells and generate “**a/a/**α/α” tetraploid progeny. The “**a/a/**α/α” tetraploid cells then undergo homozygosis at the *MTL* locus and become homozygous “α/α/α/α” cells. Tetraploid “α/α/α/α” cells mate with diploid “**a/a**” cells and generate hexaploid progeny.
(TIF)

S3 Fig Opposite-sex mating between 4N cells and 2N cells of *C*. *tropicalis*. Cells of the two mating partners were mixed and grown on Lee’s GlcNAc (pH 8.5) at 25°C for 48 hours. Mating mixtures were then plated on SCD media (-Arg, -His, or -both) with or without nourseothricin (NAT) for selectable growth. Mating progeny grown out from SCD-Arg–His plates containing nourseothricin were subject to PCR verification of the *MTL* type. (A) Mating between 2N **a** cells (**a/a**, diploid) and 4N α cells (α/α/α/α, tetraploid). Strains used: CAY3741 (2N, *MTL***a**/**a,** *his1/his1*, *arg4/arg4*, *SAT1+*), DH1185 (4N, *MTL*α/α/α/α). (B) Mating between 4N **a** cells (**a/a/a/a**, tetraploid) and 2N α cells (α/α, diploid). Strains used: DH1200 (4N, *MTL***a/a/a/a,** *arg4/arg4 SAT1+*), CAY2061 (2N, *MTL*α/α *his1/his1*). Arrow, indicates a mating conjugation. Scale bar, 10 μM. a. Cellular morphology of single cultures. b. Cellular morphology of mating mixture. c. PCR verification of *MTL* types.
(TIF)

S4 Fig Examples of YCB-BSA assays using progeny strains generated from the coupled process of same- and opposite-sex mating. The size of the BSA precipitation ring indicates the robustness of Sap activity. Progeny strains with distinct colony appearances from the “**a** x **a** + α helper” mating were examined. Diploid parental strains (CAY2060, GH1374h and CAY4149) served as controls. Cells were first grown on Lee’s GlcNAc (pH 8.5) at 25°C for seven days. 5×10^6^ cells of each strain in 5 μL ddH_2_O were spotted onto YCB-BSA plates for three days.
(TIF)

S5 Fig Genomic diversity in progeny with different morphologies generated from the coupled process of same- and opposite-sex mating. (A) Schematic diagram of morphological and genomic DNA analysis. Single colonies of mating progeny were first patched on SCD medium. Original ploidies were determined using patched cells. Patched cells were replated on Lee’s glucose medium for morphological analysis (**Fig 5**). Cells with different morphological phenotypes were grown up in liquid SCD medium for genomic DNA content analysis by flow cytometry. (B) Examples of cells from progeny with different morphologies and with higher ploidy levels (≥4N). Original ploidy (Ori. Ploidy) and colony morphologies are also indicated. This figure is related to **Fig 5**.
(TIF)

S6 Fig Genomic stability analysis of two progeny (5N and 6N) and their derived strains with distinct morphologies. (A) A schematic diagram for the experimental procedure. Cells of the original mating progeny (a) or four representative derivatives with distinct morphologies on Lee’s glucose plates (b, c, d, and e, cultured for five days at 25°C, related to **Fig 5**) were inoculated into 3 mL liquid Lee’s glucose medium (pH 6.8, 5 x 10^5^ cells/mL). After 24 hours of growth (to 3 x 10^7^ cells/mL, approximately 7 generations), cells were re-inoculated into fresh medium at the same concentration (5 x 10^5^ cells/mL). In total, 20 re-inoculations (approximately 130 generations) were performed. Cells of the original colonies, 5^th^, 15^th^, and 20^th^ inoculations were subject to FACS analysis (shown in panels **B** and **C**). (B) Dynamics of genomic DNA content in the original 5N progeny (5N-O) and its four derived strains (5N-d1 to 5N-d4). After 20 passages in liquid medium (red line), (a, 5N-O) three major ploidies (4.5N, 3N, and 2N) were observed; (b, 5N-d1), 4.8N to 4.2N; (c, 5N-d2), 4.5N to 3.2N; (d, 5N-d3), most cells were stable at 4.2N; (e, 5N-d4), 4.8N to 3N. (C) Dynamics of genomic DNA in the original 6N progeny (6N-O) and its four derived strains (6N-d1 to 6N-d4). After 20 passages in liquid medium (red line), (a, 6N-O) 6N to 2.2N; (b, 6N-d1), 4.5N to 2.5N; (c, 6N-d2), 5.3N to 4.6N and 2.5N; (d, 6N-d3), 6N to 2.2N; (e, 6N-d4), most cells were stable at 3N.
(TIF)

S1 Table Strains used in this study. (DOCX)

S2 Table Primers used in this study. (DOCX)
