# Supplementary figures and images for: A coupled process of same- and opposite-sex mating generates polyploidy and genetic diversity in Candida tropicalis
Source: PLoS Genet. 2018 May 7;14(5):e1007377. doi: 10.1371/journal.pgen.1007377 (PMC5957450; doi:10.1371/journal.pgen.1007377)

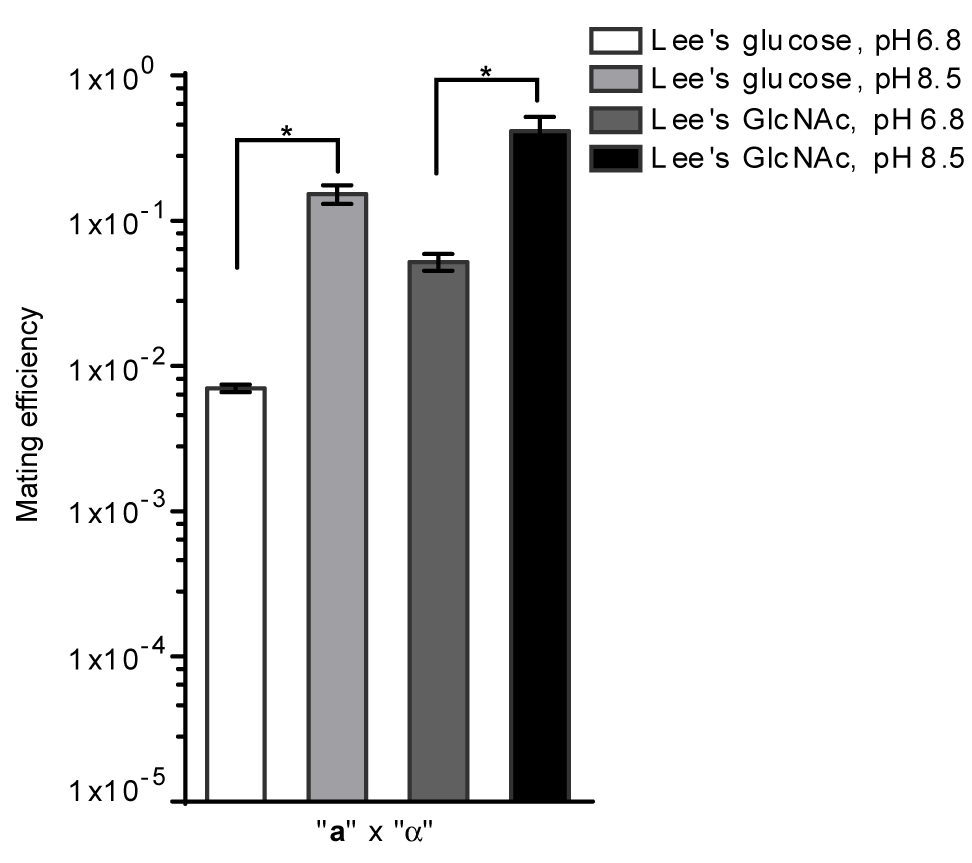

Supplement: S1 Fig — Lee’s glucose (pH 6.8 and pH 8.5) and Lee’s GlcNAc (pH 6.8 and pH 8.5) media were used. Strains used: CAY2060 (MTLa) and CAY2061 (MTLα). “a” cells (5 x10^6^) and “α” cells (5 x10^6^) were mixed and grown on different media at 25°C for seven days. Cells were then replated onto SCD media (-Arg, -His, or -both) for selectable growth of parental and mating progeny cells. Mating efficiencies were calculated according to the colony numbers obtained from SCD media. (TIF) [file pgen.1007377.s001.tif]

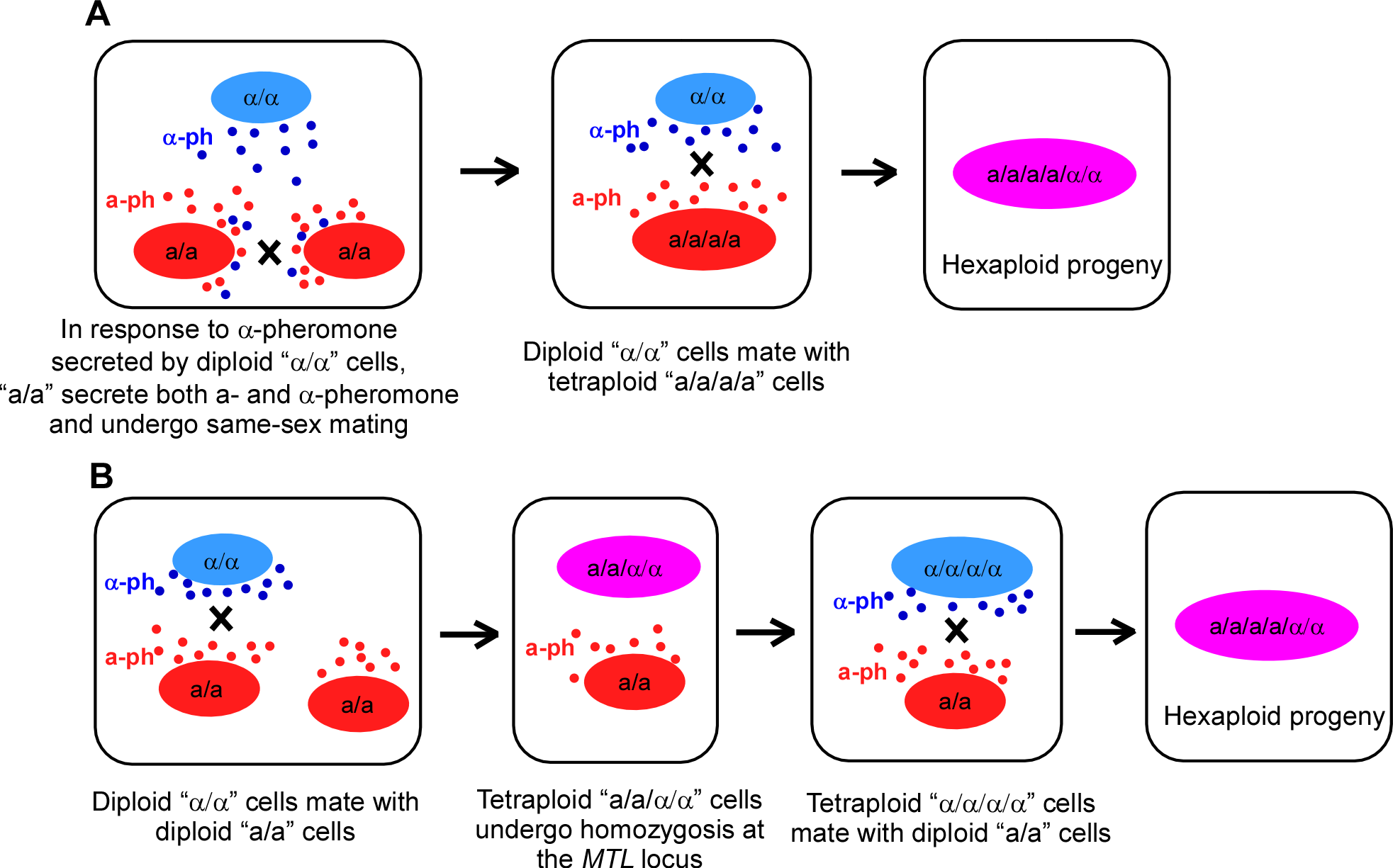

Supplement: S2 Fig — (A) Model for the same- and opposite-sex mating coupled process. Diploid “α/α” cells secrete α-pheromone and promote “a/a x a/a” same-sex mating. The tetraploid “a/a/a/a” progeny cells then mate with diploid “α/α” cells and generate hexaploid progeny. (B) Model for the two consecutive opposite-sex matings. Diploid “a/a” cells first mate with diploid “α/α” cells and generate “a/a/α/α” tetraploid progeny. The “a/a/α/α” tetraploid cells then undergo homozygosis at the MTL locus and become homozygous “α/α/α/α” cells. Tetraploid “α/α/α/α” cells mate with diploid “a/a” cells and generate hexaploid progeny. (TIF) [file pgen.1007377.s002.tif]

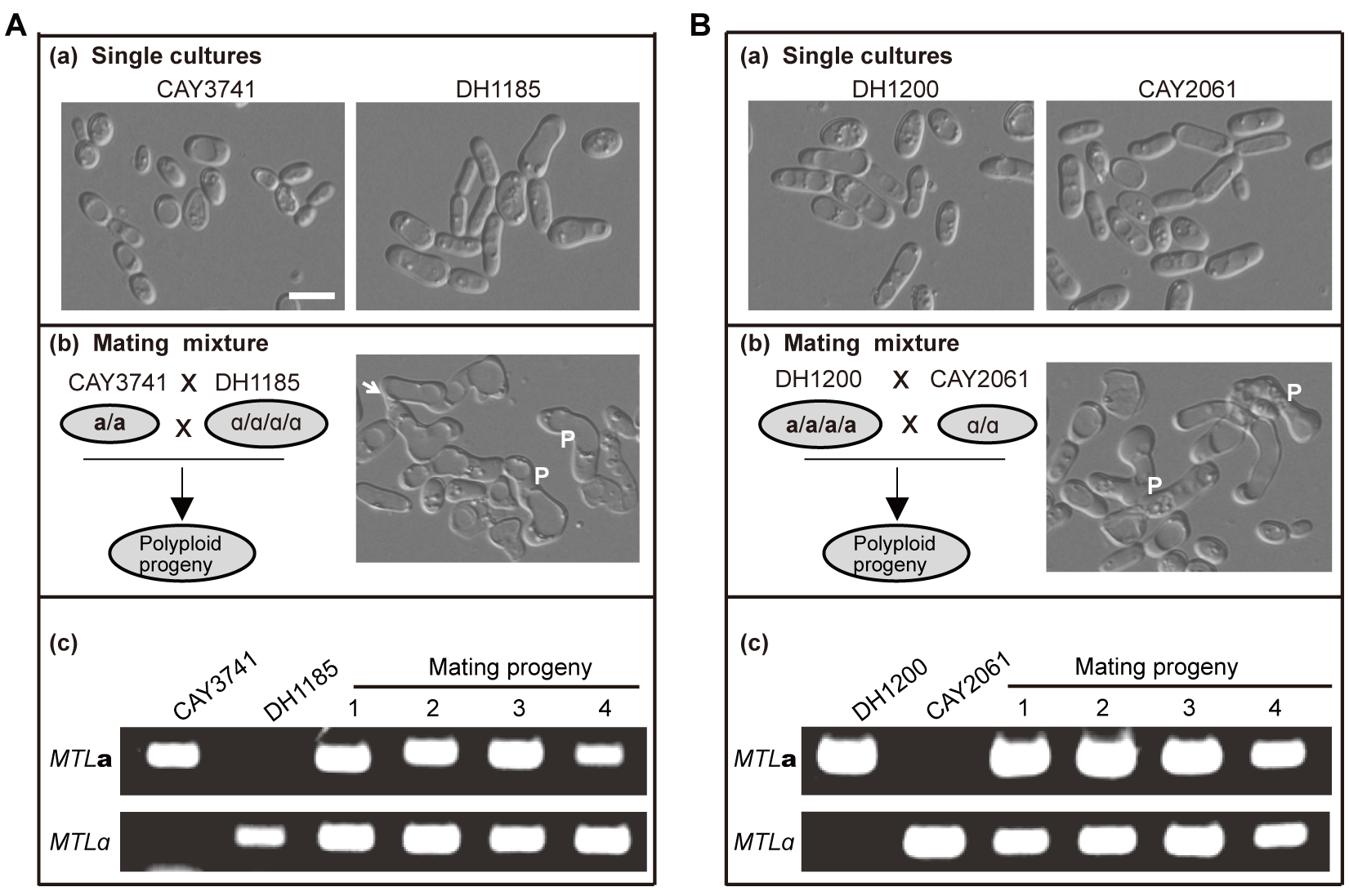

Supplement: S3 Fig — Cells of the two mating partners were mixed and grown on Lee’s GlcNAc (pH 8.5) at 25°C for 48 hours. Mating mixtures were then plated on SCD media (-Arg, -His, or -both) with or without nourseothricin (NAT) for selectable growth. Mating progeny grown out from SCD-Arg–His plates containing nourseothricin were subject to PCR verification of the MTL type. (A) Mating between 2N a cells (a/a, diploid) and 4N α cells (α/α/α/α, tetraploid). Strains used: CAY3741 (2N, MTLa/a, his1/his1, arg4/arg4, SAT1+), DH1185 (4N, MTLα/α/α/α). (B) Mating between 4N a cells (a/a/a/a, tetraploid) and 2N α cells (α/α, diploid). Strains used: DH1200 (4N, MTLa/a/a/a, arg4/arg4 SAT1+), CAY2061 (2N, MTLα/α his1/his1). Arrow, indicates a mating conjugation. Scale bar, 10 μM. a. Cellular morphology of single cultures. b. Cellular morphology of mating mixture. c. PCR verification of MTL types. (TIF) [file pgen.1007377.s003.tif]

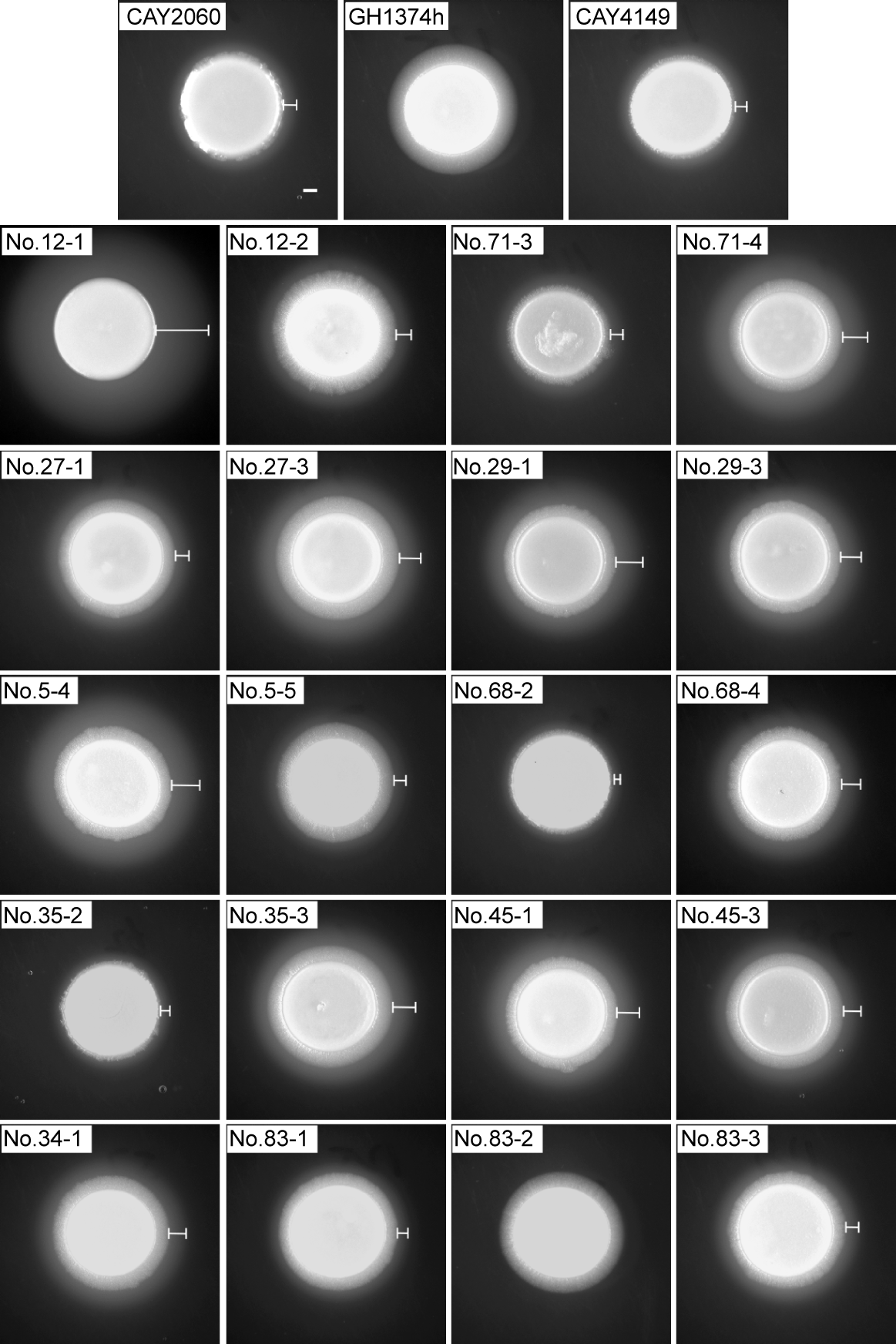

Supplement: S4 Fig — The size of the BSA precipitation ring indicates the robustness of Sap activity. Progeny strains with distinct colony appearances from the “a x a + α helper” mating were examined. Diploid parental strains (CAY2060, GH1374h and CAY4149) served as controls. Cells were first grown on Lee’s GlcNAc (pH 8.5) at 25°C for seven days. 5×10^6^ cells of each strain in 5 μL ddH2O were spotted onto YCB-BSA plates for three days. (TIF) [file pgen.1007377.s004.tif]

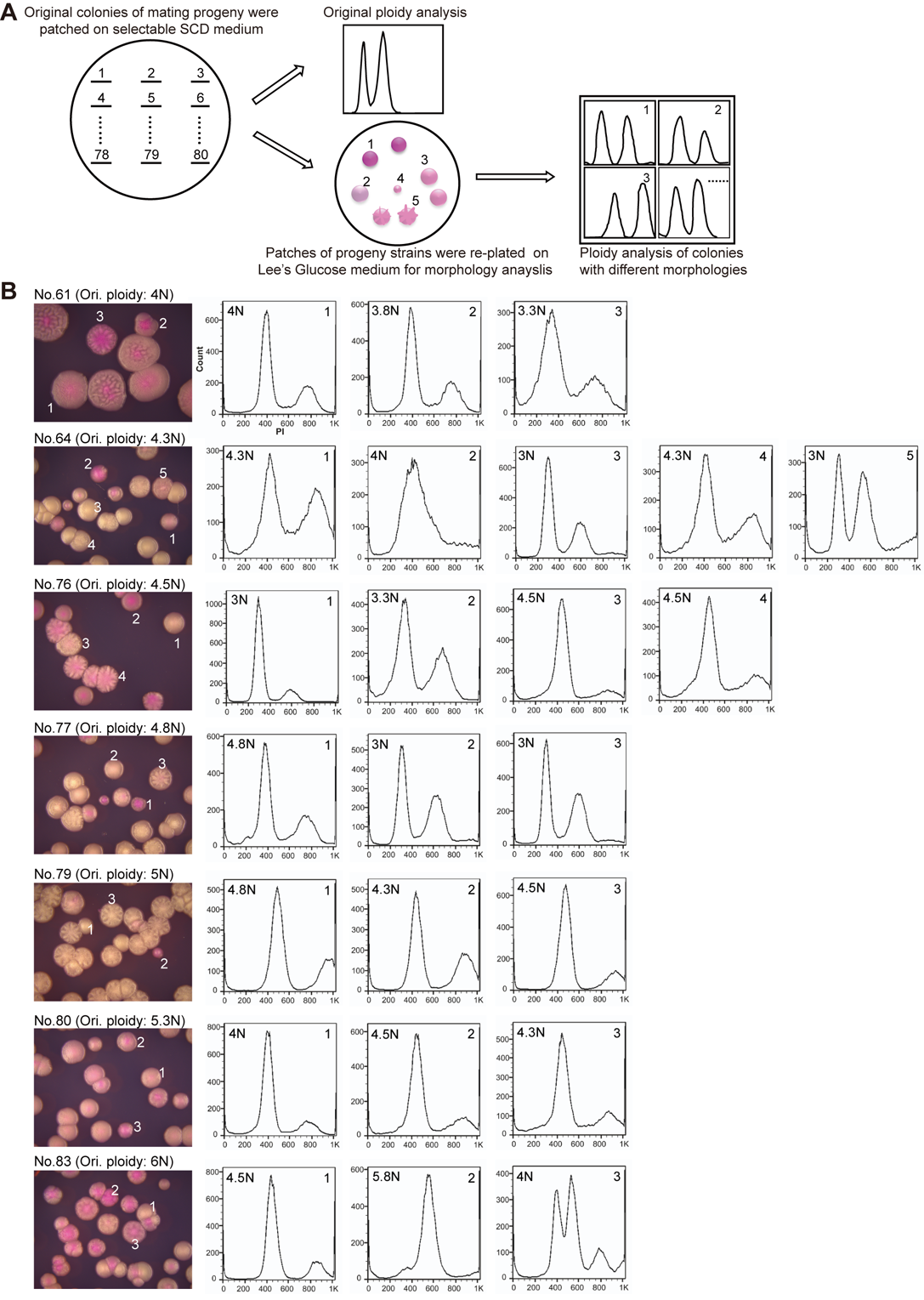

Supplement: S5 Fig — (A) Schematic diagram of morphological and genomic DNA analysis. Single colonies of mating progeny were first patched on SCD medium. Original ploidies were determined using patched cells. Patched cells were replated on Lee’s glucose medium for morphological analysis (Fig 5). Cells with different morphological phenotypes were grown up in liquid SCD medium for genomic DNA content analysis by flow cytometry. (B) Examples of cells from progeny with different morphologies and with higher ploidy levels (≥4N). Original ploidy (Ori. Ploidy) and colony morphologies are also indicated. This figure is related to Fig 5. (TIF) [file pgen.1007377.s005.tif]

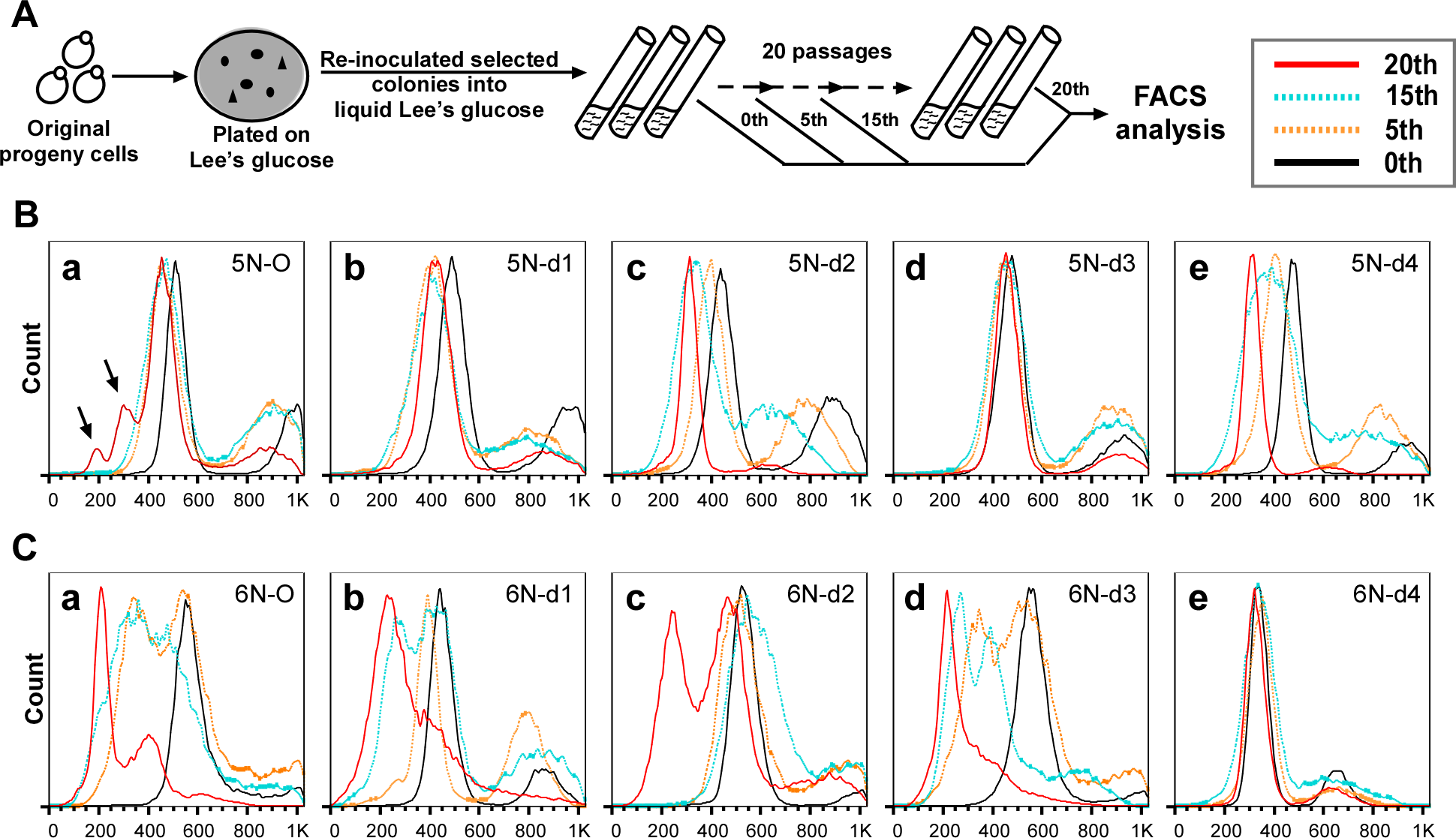

Supplement: S6 Fig — (A) A schematic diagram for the experimental procedure. Cells of the original mating progeny (a) or four representative derivatives with distinct morphologies on Lee’s glucose plates (b, c, d, and e, cultured for five days at 25°C, related to Fig 5) were inoculated into 3 mL liquid Lee’s glucose medium (pH 6.8, 5 x 10^5^ cells/mL). After 24 hours of growth (to 3 x 10^7^ cells/mL, approximately 7 generations), cells were re-inoculated into fresh medium at the same concentration (5 x 10^5^ cells/mL). In total, 20 re-inoculations (approximately 130 generations) were performed. Cells of the original colonies, 5th, 15th, and 20th inoculations were subject to FACS analysis (shown in panels B and C). (B) Dynamics of genomic DNA content in the original 5N progeny (5N-O) and its four derived strains (5N-d1 to 5N-d4). After 20 passages in liquid medium (red line), (a, 5N-O) three major ploidies (4.5N, 3N, and 2N) were observed; (b, 5N-d1), 4.8N to 4.2N; (c, 5N-d2), 4.5N to 3.2N; (d, 5N-d3), most cells were stable at 4.2N; (e, 5N-d4), 4.8N to 3N. (C) Dynamics of genomic DNA in the original 6N progeny (6N-O) and its four derived strains (6N-d1 to 6N-d4). After 20 passages in liquid medium (red line), (a, 6N-O) 6N to 2.2N; (b, 6N-d1), 4.5N to 2.5N; (c, 6N-d2), 5.3N to 4.6N and 2.5N; (d, 6N-d3), 6N to 2.2N; (e, 6N-d4), most cells were stable at 3N. (TIF) [file pgen.1007377.s006.tif]
