# Supplementary material for: A coupled process of same- and opposite-sex mating generates polyploidy and genetic diversity in Candida tropicalis
Source: PLoS Genet. 2018 May 7;14(5):e1007377. doi: 10.1371/journal.pgen.1007377 (PMC5957450; doi:10.1371/journal.pgen.1007377)
Supplement: S1 Table — (DOCX) [file pgen.1007377.s007.docx]

**Table S1. Strains used in this study.**

| **Strain name** | **Parent strain** | **Genotype or description** | **Ploidy** | **Reference** |
| --- | --- | --- | --- | --- |
| JX1016 |  | Clinical isolate, *MTL***a**/*ɑ* | 2N | [1] |
| GH1374h | GH1374 | Clinical isolate, *MTL****a****/****a,*** *his1/his1* | 2N | [2] |
| CAY2060 | ATCC 34139 ST-120 | *arg4/arg4*, *MTL***a/a** | 2N | [3] |
| CAY2061 | ATCC 34139 ST-120 | *his1/his1*, *MTLɑ/ɑ* | 2N | [3] |
| CAY2063 | ATCC 34139 ST-120 | *arg4/arg4, MTLɑ/ɑ* | 2N | [3] |
| CAY4149 |  | *arg4/arg4, his1/his1,MTLɑ/ɑ* | 2N | [3] |
| CAY3741 |  | *arg4/arg4, his1/his1, MTL***a**/**a,** *SAT1* | 2N | [3] |
| CAY2200 | ATCC 34139 ST-120 | *his1/his1 ste2/ste2, MTL***a***/***a** | 2N | [3] |
| CAY2202 | ATCC 34139 ST-120 | *arg4/arg4 ste2/ste2,MTL***a***/***a** | 2N | [3] |
| CAY2246 | ATCC 34139 ST-120 | *arg4/arg4 ste2/ste2/STE2::SAT1,MTL***a***/***a** | 2N | [3] |
| CAY2247 | ATCC 34139 ST-120 | *his1/his1 ste2/ste2/STE2::SAT1,MTL***a***/***a** | 2N | [3] |
| CAY2205 | ATCC 34139 ST-120 | *arg4/arg4 wor1/wor1::SAT1 MTLa/a* | 2N | [3] |
| CAY2342 | ATCC 34139 ST-120 | *his1/his1 wor1/wor1::SAT1 MTL α/α* | 2N | [3] |
| DH1175 |  | *MTL***a***/α,* opposite mating product (CAY2060 x CAY2061) | 4N | This study |
| DH1185 |  | *MTLα,* three-way mating product (CAY2061 x CAY2063 + CAY3741) | 4N | This study |
| DH1200 |  | *MTL***a,** *arg4- SAT1*+*,* three-way mating product (CAY2060 x CAY3741+ CAY4149) | 4N | This study |
| DH1210 |  | *MTL***a**, mating product of CAY2060 x GH1374 in the presence of α-factor | 4N | This study |
| DH1211 |  | *MTL***a**, mating product of CAY2060 x GH1374 in the presence of α-factor | 4N | This study |
| DH1246 | CAY3741 | *arg4/arg4 his1/HIS1,MTL***a***/***a** | 2N | This study |
| DH1249 | CAY3741 | *arg4/ARG4 his1/his1,MTL***a***/***a** | 2N | This study |
| DH1251 | CAY4149 | *arg4/arg4 his1/ HIS1,MTLα/α* | 2N | This study |
| DH1254 | CAY4149 | *arg4/ ARG4 his1/his1,MTLα/α* | 2N | This study |
| DH1259 | DH1249 | *arg4/ARG4 his1/HIS1,MTL***a***/***a** | 2N | This study |
| DH1271 |  | *MTL***a***/α,* three-way mating product (DH1251 x DH1254 + CAY3741) | 3N | This study |
| DH1276 |  | *MTL***a***/α,* three-way mating product (DH1251 x DH1254 + CAY3741) | 5N | This study |
| DH1286 |  | *MTL***a***/α,* three-way mating product (DH1251 x DH1254 + CAY3741) | 4N | This study |

1. Xie J, Du H, Guan G, Tong Y, Kourkoumpetis TK, Zhang L, Bai FY, Huang G. N-acetylglucosamine induces white-to-opaque switching and mating in Candida tropicalis, providing new insights into adaptation and fungal sexual evolution. Eukaryot Cell. 2012,11(6):773-82.
2. Zhang Q, Tao L, Guan G, Yue H, Liang W, Cao C, Dai Y, Huang G. Regulation of filamentation in the human fungal pathogen Candida tropicalis. Mol Microbiol. 2016, 99(3):528-45.
3. Porman AM, Alby K, Hirakawa MP, Bennett RJ. Discovery of a phenotypic switch regulating sexual mating in the opportunistic fungal pathogen Candida tropicalis. Proc Natl Acad Sci U S A. 2011, 108(52):21158-63.
