# Supplementary material for: A coupled process of same- and opposite-sex mating generates polyploidy and genetic diversity in Candida tropicalis
Source: PLoS Genet. 2018 May 7;14(5):e1007377. doi: 10.1371/journal.pgen.1007377 (PMC5957450; doi:10.1371/journal.pgen.1007377)
Supplement: S2 Table — (DOCX) [file pgen.1007377.s008.docx]

| **Table S2. Primers used in this study.** | | | |
| --- | --- | --- | --- |
| **No.** | **Name** | **Sequence (5' to 3')** | **Purpose** |
| 1 | CtA1-F101 | AGGCAGATGATTTTCGAGCC | for verification of MTL**a**1 in C. tropicalis |
| 2 | CtA1-R424 | ATTCCTTCTTGGAAGAATCCGGT |  |
| 3 | CtAlpha2-F12 | CTGGGATACCCTTCACCAAGTC | for verification of MTLɑ2 in C. tropicalis |
| 4 | CtAlpha2-R400 | TCTGGAACCACCCTTCCAAT |  |
| 5 | CtARG4(5’783) | GGGTAGAAAGCTGCTGGACT | for amplification of C. tropicalis *ARG4* |
| 6 | CtARG4(3’719) | TCATACTGAACACCCGTGGC |  |
| 7 | CtHIS1(5’605) | TGGGTATGGCATCAGCAACAA | for amplification of C. tropicalis HIS1 |
| 8 | CtHIS1(3’334) | CTCAATTGGCCACATCCCTC |  |
| 9 | CtSTE2F136 | GTCATGGGTGTGATGATTGG | for STE2 qPCR |
| 10 | CtSTE2R312 | GGAAAAACAAGCAGCCCGAA |  |
| 11 | CtSTE3F20 | GCATGGGTGCCCTTTTGTTT | for STE3 qPCR |
| 12 | CtSTE3R202 | CAATGCCATCCCAGGAAGT |  |
| 13 | CtMFalphaF218 | CTGCTGTTGAAGAAGCCTAC | for MFɑ qPCR |
| 14 | CtMFalphaR369 | GGCGTCAATCTCTTCTCTCT |  |
| 15 | MFa1F1 | ATGGCTGCTCAACAATCTAACA | for MFa2 qPCR |
| 16 | MFa1R120 | ACAATTGGAAGCCATGTATCCT |  |
| 17 | ctACT1F | TGAGATACCCAATTGAACAC | for ACT1 qPCR |
| 18 | ctACT1R | ACCATCACCAGAATCCAAGAC |  |
